# Emerging 2D Materials and Their Hybrid Nanostructures for Label-Free Optical Biosensing: Recent Progress and Outlook

**DOI:** 10.1002/adfm.202513767

**Published:** 2025-09-25

**Authors:** Xinyi Li, Yonghao Fu, Yuehe Lin, Dan Du

**Affiliations:** School of Mechanical and Material Engineering, Washington State University, Pullman, WA 99164, USA; School of Mechanical and Material Engineering, Washington State University, Pullman, WA 99164, USA; School of Mechanical and Material Engineering, Washington State University, Pullman, WA 99164, USA; School of Mechanical and Material Engineering, Washington State University, Pullman, WA 99164, USA; Department of Pharmaceutical Sciences, College of Pharmacy and Pharmaceutical Sciences, Spokane, WA 99202, USA

**Keywords:** 2D materials, biosensors, hybrid nanostructures, surface plasmon resonance, surface-enhanced Raman scattering

## Abstract

The increasing demand for rapid detection and remote monitoring with high sensitivity and selectivity has driven the development of label-free optical biosensors based on 2D materials (2DMs). Owing to their high surface-to-volume ratio and unique, tunable electrical and optical properties, 2DMs are highly attractive for biosensing applications. Van der Waals heterostructures (vdWHs) that combine different 2DMs allow the creation of hybrid materials with synergistic or novel functionalities. Beyond purely 2D vdWHs, mixed-dimensional hybrid architectures have recently emerged, offering enhanced sensitivity, stability, and reproducibility in label-free optical detection. This review highlights recent advances in surface-enhanced Raman scattering (SERS)- and surface plasmon resonance (SPR)-based biosensing using 2DMs and their hybrid nanostructures, discussing the role of each component and emphasizing their synergistic effects. It also provides an overview of recent progress in multifunctional sensing platforms and the integration of machine learning to improve performance and enable intelligent data analysis. Finally, the current challenges and future opportunities for advancing next-generation label-free optical biosensors are highlighted.

## 1. Introduction

**Figure 1.**
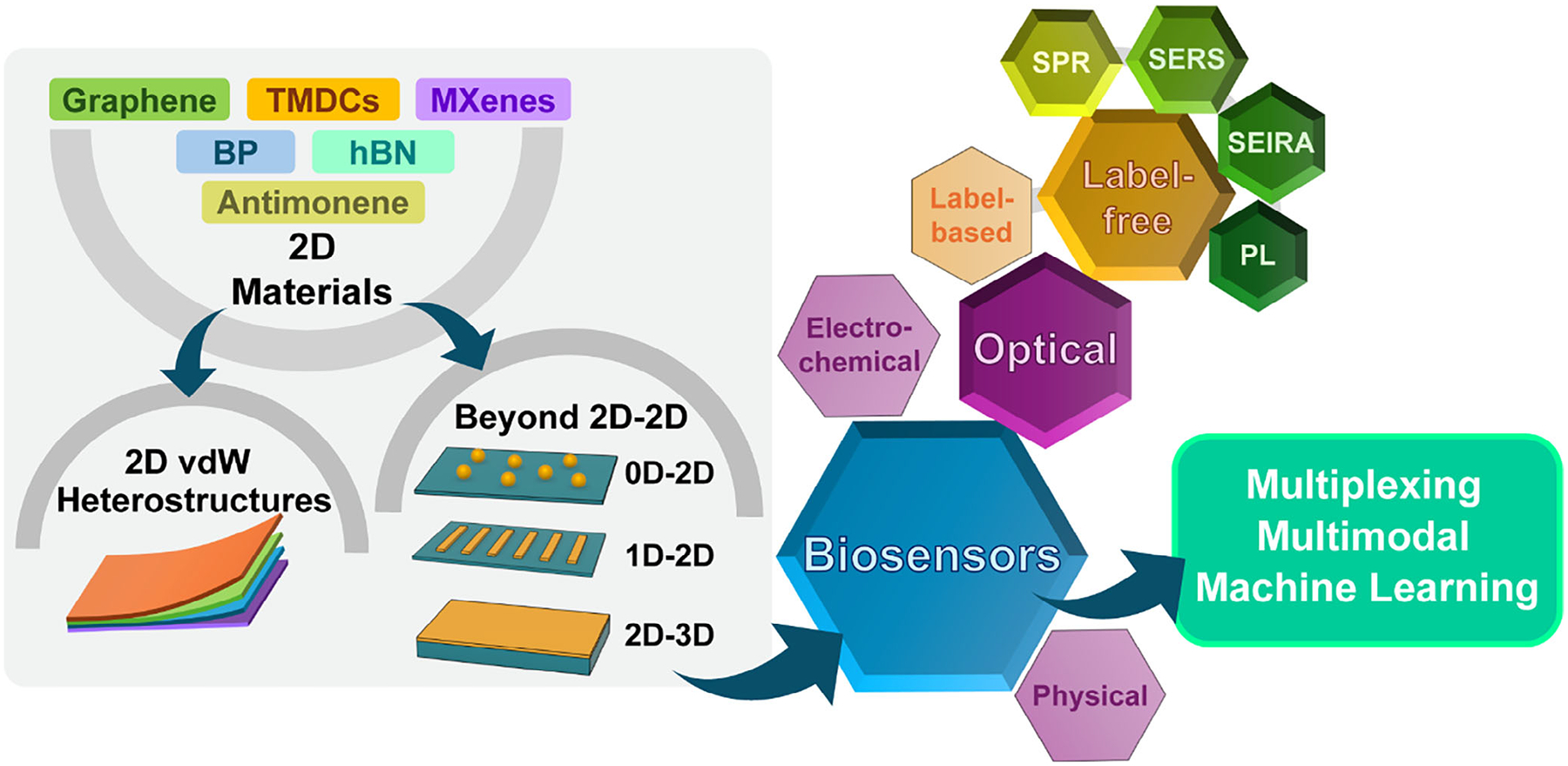
2DMs and their mixed-dimensional hybrid nanostructures for label-free optical biosensing applications.

Nowadays, growing public concern about healthcare and environmental conditions has driven rapid advancements in biosensor technologies, particularly those delivering high sensitivity, fast response, and real-time monitoring capabilities.^[1–6]^ Based on the type of transducer, biosensors are classified into three major categories^[7]^: electrochemical, physical, and optical (Figure 1). Among them, optical biosensors have become increasingly attractive, owing to their non-invasive nature, reliable performance in harsh environments, and capability for multiplexed analyte detection.^[8–10]^

Although label-based optical biosensing, which employs fluorescent dyes or other tags on target biomolecules, can achieve high sensitivity down to the single-molecule level,^[11,12]^ it presents notable limitations. Primarily, the presence of labels may disrupt biomolecular function. Furthermore, background fluorescence from unbound labels can introduce signal bias, thereby compromising analytical accuracy, particularly when detecting analytes at ultralow concentrations.^[7]^ In contrast, label-free biosensing neither requires external labels nor relies on their properties, facilitating accurate quantitative analysis of biomolecule detection and interactions. Additionally, label-free approaches are capable of detecting a wide variety of bioanalytes that are difficult to tag or lack known receptors.^[7,13,14]^ Label-free optical biosensors, in particular, offer several advantages over other label-free techniques, including high stability and specificity, multiplexing and real-time monitoring capabilities, and high throughput. These features, combined with their broad applicability across medical diagnostics, environmental monitoring, food safety, and drug development, have driven intensive research efforts into their design and advancement.^[14–17]^

As biosensor technology progresses, there is an increasing demand for platforms that are not only stable, reliable, and selective but also compact, flexible, and highly sensitive. This is where 2D materials (2DMs) play a key role.^[18–25]^ Their atomically thin nature contributes to the compactness and flexibility of biosensors, while their high surface-to-volume ratio boosts sensing efficiency, enabling ultra-sensitive detection of bioanalytes.^[26,27]^ Moreover, 2DMs exhibit unique electronic and optical properties absent in their bulk counterparts, such as tunable bandgaps, strong light–matter interactions, and strain engineering.^[28–30]^ These features have been extensively explored for label-free biosensing applications. For instance, 2DMs have been used in fabricating field-effect transistor (FET) biosensors, demonstrating their potential in point-of-care diagnostics.^[31–33]^ In particular, 2DMs have shown great promise in advancing label-free optical biosensing platforms, including surface plasmon resonance (SPR)^[34–36]^ and surface-enhanced Raman scattering (SERS) sensors.^[37–39]^

To further tailor the properties of 2DM-based biosensors, researchers have explored stacking different 2DMs into 2D van der Waals heterostructures (vdWHs), often described as “Lego-like” assemblies (Figure 1). These intentionally designed vdWHs combine the properties of multiple 2DMs, enabling more complex functionalities and greater tunability, thereby surpassing the capabilities of single-material systems.^[40–42]^ This strategy also supports device miniaturization, aligning with the trend toward compact biosensors. Notably, vdWH-based biosensors can offer multimodal detection—providing multiple types of signals in response to the presence of analytes—which allows for cross-validation and improves the sensing accuracy. Moreover, vdWH-based sensors are capable of multiplexed detection of various analytes within a complex biological matrix, significantly improving sensing throughput.

Beyond 2D-2D vdWHs, integrating 2DMs with 0D (e.g., nanoparticles (NPs), quantum dots (QDs)) and 1D (e.g., nanowires (NWs), nanorods (NRs), nanoribbons) nanostructures^[43–45]^ further diversifies biosensor functionality by exploiting confinement across multiple dimensions. Mixed-dimensional hybrid nanostructures offer enhanced performance—including ultra-high sensitivity, real-time monitoring, multiplexed detection, and device miniaturization^[46,47]^—paving the way toward realizing the “lab-on-a-chip” vision.^[48,49]^

So far, several reviews have discussed the properties of 2DMs and their label-free optical biosensing applications. However, no existing review comprehensively addresses both 2DMs and their advanced hybrid nanostructures for label-free optical biosensing, emphasizing the unique roles of individual 2DMs along with the synergistic or novel functionalities arising from their nanoarchitectures. Given the growing demand for compact, high-performance, multifunctional biosensors in healthcare and environmental monitoring, it becomes essential to integrate the advantages of various low-dimensional materials. For this purpose, designing mixed-dimensional nanostructures has emerged as a compelling strategy to expand material properties and introduce novel functionalities.

In this review, we summarize the role of 2DMs and their hybrid nanostructures in advancing label-free optical biosensing, emphasizing recent progress from the perspective of their synergistic effects within nanohybrids. We begin with a concise introduction to label-free optical biosensing techniques, including SPR, SERS, surface-enhanced infrared absorption (SEIRA), and photoluminescence (PL) sensing. Next, representative 2DMs—such as graphene, transition metal dichalcogenides (TMDCs), black phosphorus (BP), hexagonal boron nitride (hBN), MXenes, and antimonene—are briefly described, focusing on their optical biosensing-relevant properties. Subsequently, we discuss mixed-dimensional hybrid nanostructures based on 2DMs, their example applications in label-free optical biosensing, and provide a brief overview of their synthesis and fabrication techniques. In particular, we highlight recent advances in emerging 2DMs and their hybrids for SERS- and SPR-based biosensing, as well as the development of multifunctional platforms. Finally, we outline current challenges and future directions, including performance and stability optimization, advanced data analysis using machine learning (ML), and the importance of interdisciplinary research in realizing next-generation biosensors. We anticipate that this review will provide a valuable reference for researchers in optical biosensing and offer timely insights into the design and development of advanced 2DM- and hybrid nanostructure-based sensing platforms.

## 2. Representative Label-Free Optical Biosensing Techniques

**Figure 2.**
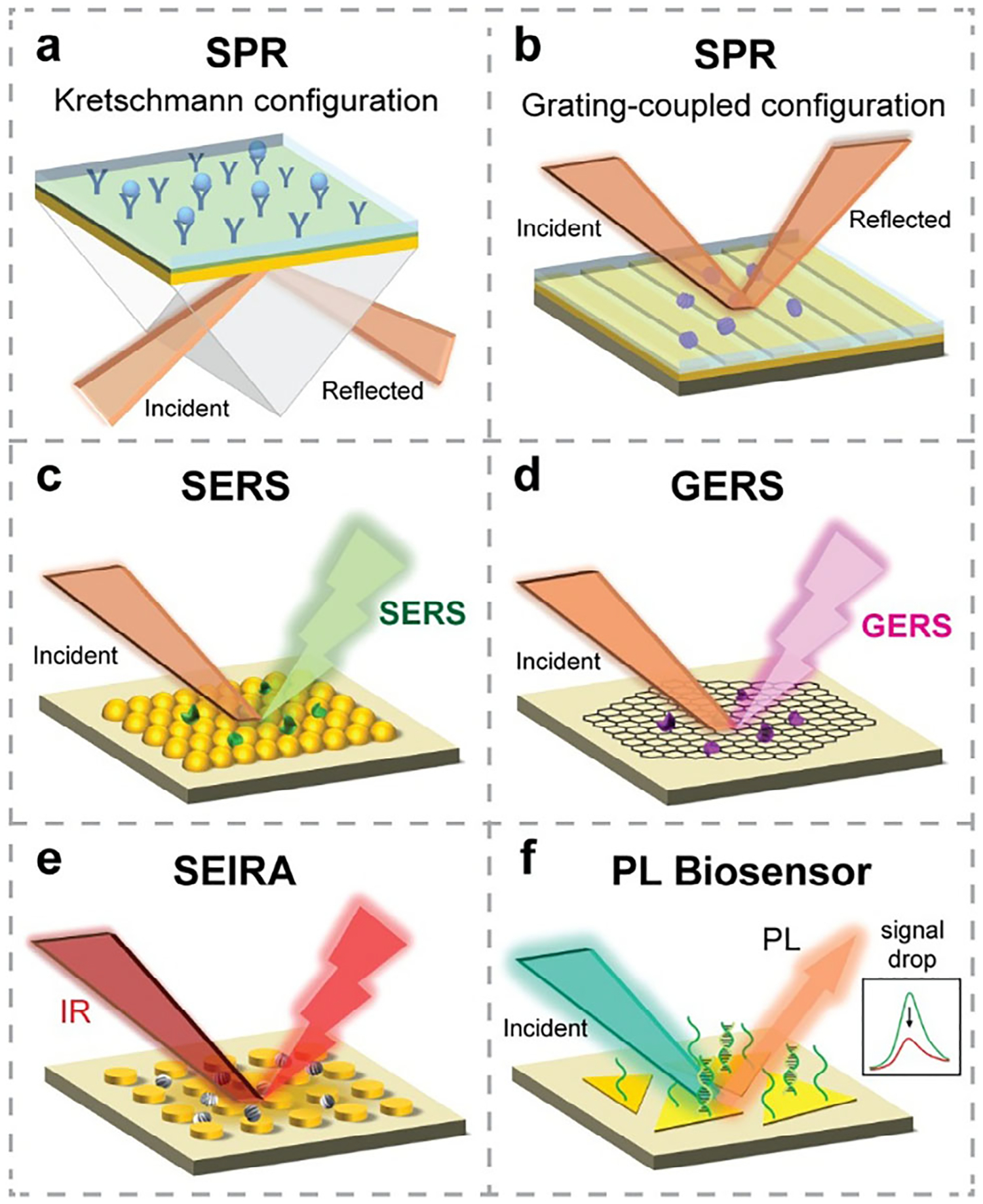
Representative label-free optical biosensing techniques. a) SPR with Kretschmann configuration. b) SPR with grating-coupled configuration. c) SERS. d) GERS. e) SEIRA. f) PL biosensor.

Label-free optical biosensing is a class of biosensing technologies that rely on changes in optical parameters—such as refractive index (RI), Raman scattering, fluorescence, and absorption—as transduction mechanisms for detecting bioanalytes.^[7]^ Overcoming the drawbacks of fluorescent labels, label-free approaches enable real-time and continuous monitoring of biomolecular interactions while preserving the native function of biomolecules. This enhances the reliability and reproducibility of measurements by minimizing interference from labeling agents and supports the development of miniaturized and multiplexed sensing platforms. Representative examples of label-free optical biosensors include SPR, SERS, SEIRA, and PL biosensors, as illustrated in Figure 2.

### 2.1. SPR Biosensors

SPR biosensors have been widely applied in various fields, including life sciences, food safety, medical diagnostics, and drug delivery.^[50]^ The working principle of SPR biosensors is based on the excitation of surface plasmons at the metal-dielectric interface, typically supported by Au or Ag thin film. This excitation produces an evanescent field that is highly sensitive to RI changes near the surface, enabling the detection of binding events with minimal interference from unbound analytes. Under resonant conditions, the reflected light intensity exhibits a sharp dip, and the corresponding incident angle is referred to as the SPR angle.^[35]^ Binding events at the sensor surface alter the local dielectric function, resulting in a shift in the SPR angle. Recording this measurable shift enables real-time, label-free detection of interactions such as antibody-antigen binding, nucleic acid hybridization, and protein-ligand interactions.

To excite surface plasmons, both energy and in-plane momentum must be matched. For this purpose, coupling elements such as prisms or gratings are often employed. Accordingly, various SPR biosensor configurations have been developed, including the commonly used Kretschmann configuration (Figure 2a), Otto configuration, grating-coupled structures (Figure 2b), and fiber-optic-based setups.

### 2.2. SERS

SERS offers a sensitive and non-destructive approach for label-free optical biosensing (Figure 2c). It is rapid and low-cost compared to traditional analytical techniques.^[37]^ The inherent selectivity required for biosensing is naturally met by Raman scattering, as Raman spectra serve as unique molecular fingerprints. By utilizing metal NPs (e.g., Au NPs, Ag NPs, etc.), the Raman signal can be significantly enhanced when molecules are adsorbed in the vicinity—i.e., at “hot spots” around the NPs—due to the strong localized electromagnetic (EM) fields. This enhancement enables highly sensitive, label-free detection of biomolecules, pathogens, and chemical analytes, making SERS a powerful tool in optical biosensing. Compared with other label-free optical biosensing techniques—for example, PL biosensors—SERS excels in its multiplexed detection capability and high stability.

Recently, SERS substrates based on 2DMs have been developed to overcome the limitations of traditional SERS platforms. In 2DM-based SERS systems, Raman signal amplification is attributed to the chemical mechanism (CM), influenced by the electronic structure of the target analytes and the 2DM interface.^[46]^ In the case of graphene-enhanced Raman scattering (GERS) (Figure 2d), the high electrical conductivity of graphene facilitates both CM and EM enhancement effects, working synergistically to enable highly sensitive and specific detection in Raman spectroscopy.^[51]^

### 2.3. SEIRA

Similar to SERS, SEIRA is another analytical vibrational spectroscopy technique that leverages localized EM enhancement.^[52]^ Typically, metallic NPs and nanostructure arrays^[53]^ are employed as substrates, increasing the effective IR absorption cross-section of the probing molecules (Figure 2e). Recently, low-dimensional vdW materials have emerged for sensitive IR sensing, including graphene,^[54]^ carbon nanotubes,^[55]^ graphene nanoribbons,^[56,57]^ and hBN nanoresonators.^[58–60]^ While both SERS and SEIRA provide molecular fingerprints and can exhibit high specificity in biosensing, SEIRA probes IR-active (dipole-active) vibration modes, which are inherently more responsive to molecular structure than Raman-active modes.^[52,61]^ Although the intrinsic IR absorption cross-sections are larger than Raman scattering cross-sections, the EM enhancement achievable in SERS is usually significantly larger, leading to stronger overall signal enhancement and single-molecule detection capability.^[52,62]^ However, due to its tighter field confinement, SERS is less effective for probing large biomolecules, as the field enhancement is only sustained within a few nanometers of the substrate surface. In contrast, the enhanced field in SEIRA extends up to ~100 nm, enabling the detection of large biomolecules, including viruses and bacteria.^[62]^ Therefore, the choice between SERS and SEIRA depends on the size and nature of the target analytes. Overall, SEIRA offers label-free, molecular-specific detection with high sensitivity, and is well-suited for real-time analysis of biomolecular interactions, thin films, and surface chemistry.

### 2.4. PL Biosensors

PL biosensors are a type of optical biosensing platform based on changes in the PL properties of the sensing material upon interaction with target bioanalytes (Figure 2f). In this context, the sensing materials are often semiconductors—such as QDs, TMDCs, etc.—that can be excited by light and exhibit PL behavior. The presence of bioanalytes can modulate the PL response—typically through quenching or spectral shifts—via charge transfer mechanisms.^[63]^ Unlike traditional fluorescent biosensors that rely on labeled probes, PL biosensors often enable label-free detection by leveraging the intrinsic optical response of the sensing material itself. Owing to their high sensitivity, tunable emission, and compatibility with miniaturized platforms, PL biosensors are widely explored for the detection of biomolecules, DNA, RNA, metal ions, and environmental pollutants.

## 3. Typical 2DMs for Label-Free Optical Biosensing

2DMs have gained tremendous attention recently due to their extraordinary properties compared to their 3D bulk counterparts.^[64]^ They are particularly favorable for biosensing applications.^[20,32,65]^ For instance, their atomically thin nature enables mechanical flexibility and high strain tolerance, which has advanced the development of wearable and implantable biosensors,^[66–68]^ as well as the miniaturization of biosensing devices.^[48]^ More importantly, their extremely large surface area provides numerous active sites, significantly improving the sensitivity of biosensors. The large surface also allows for surface modification and functionalization, which can be tailored to enhance the selectivity of the biosensor toward different bioanalytes. Additionally, the broad range of electronic properties exhibited by 2DMs—such as tunable bandgaps, confined polaritons, and fluorescence quenching—also plays a critical role in biosensor development. In this section, we introduce several 2DMs commonly used for label-free optical biosensing, highlighting their relevant properties and representative applications (see Table 1).

### 3.1. Graphene

Graphene, the most prominent member of the carbon-based 2DMs family, is composed of a hexagonal lattice of *sp*^*2*^ carbon atoms.^[24]^ It exhibits several remarkable properties, including high tensile strength, excellent thermal conductivity, outstanding intrinsic carrier mobility, and high transparency.^[28,42]^ These attributes have enabled the widespread application of graphene across various fields, such as nanoelectronics, supercapacitors, optoelectronic devices, bioimaging, and biosensors.^[66,93]^

As a semi-metal with a zero bandgap, graphene possesses unique plasmonic properties that can enhance biosensor performance. It supports IR plasmons with high tunability, strong field confinement, long lifetimes, and low loss—features highly advantageous for label-free optical biosensing. For instance, the local RI at the sensor interface can be significantly modified in the presence of graphene, resulting in larger shifts of the SPR angle and thereby enhancing the sensitivity of SPR biosensors.^[69]^ In SEIRA-based biosensors, graphene enhances IR absorption, leading to improved sensor performance with a lower limit of detection (LOD).^[94,95]^ Notably, graphene plays a key role in the development of GERS, enabling ultrahigh sensitivity in bioanalyte detection. The enhancement in GERS primarily arises from charge transfer between graphene and adsorbed molecules, facilitated by strong *π*–*π* interactions.^[96]^

**Figure 3.**
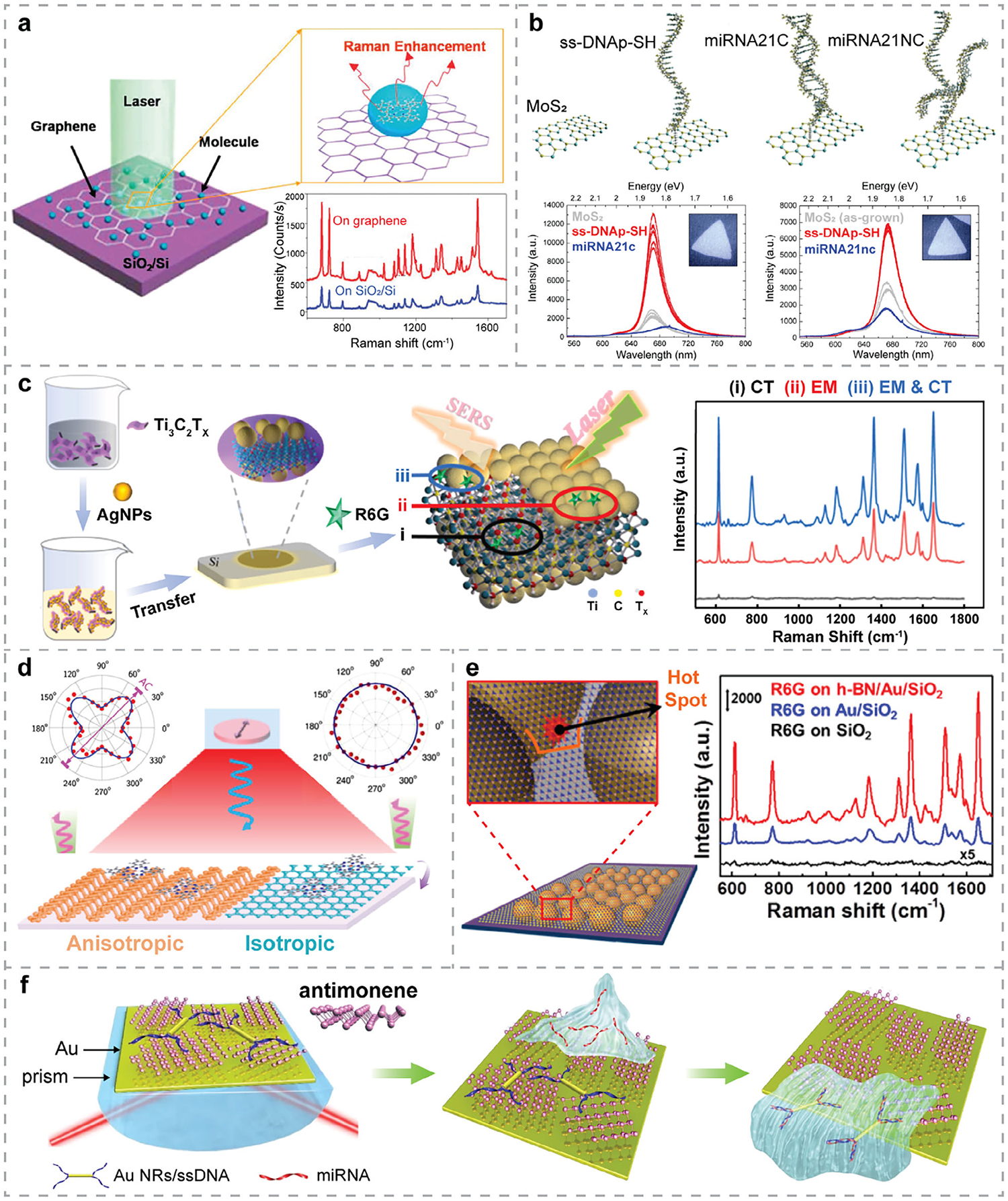
Typical 2DMs and their applications in label-free optical biosensing. a) Graphene used as a substrate for Raman enhancement. Reproduced with permission.^[97]^ Copyright 2010, American Chemical Society. b) Detection of breast cancer biomarkers via the PL of epitaxial monolayer MoS_2_ flakes. Reproduced with permission.^[72]^ Copyright 2020, Springer Nature. c) Synergistic CM and EM enhancements in a Ti_3_C_2_T_x_/Ag NP-based SERS substrate for ultrasensitive detection. Reproduced with permission.^[100]^ Copyright 2024, Elsevier. d) Observed anisotropic Raman enhancement with angular dependence on few-layer BP for detecting CuPc molecules. Reproduced with permission.^[81]^ Copyright 2015, American Chemical Society. e) Designed hBN/Au NPs substrate for manipulating surface plasmons and enhancing SERS performance. Reproduced with permission.^[87]^ Copyright 2016, American Chemical Society. f) Ultrasensitive detection of miRNA enabled by an antimonene-based SPR biosensor. Reproduced with permission.^[88]^ Copyright 2019, Springer Nature.

The concept of using graphene as a SERS substrate was first explored and validated in 2009, as reported by Ling et al.^[97]^ They conducted a series of experiments comparing the Raman signals of deposited molecules—phthalocyanine (Pc), rhodamine 6G (R6G), protoporphyrin IX, and crystal violet (CV)—on Si/SiO_2_ substrates versus graphene (Figure 3a). The resulting Raman spectra, as shown in Figure 3a, exhibited strong enhancement in the presence of monolayer graphene. Since then, GERS has developed rapidly and has been widely adopted for biosensing applications.^[51]^ Additionally, the GERS effect can be modulated by tuning the Fermi level of graphene—either through electric gating, as demonstrated by adjusting the applied voltage in graphene FETs,^[98]^ or through defect engineering, such as nitrogen doping, as performed by Feng et al.^[99]^ Furthermore, graphene’s excellent biocompatibility and ease of surface functionalization further support its integration into a wide range of biosensing platforms for the detection of glucose, DNA/RNA, bacteria, etc.

The successful exfoliation of graphene from graphite in 2004^[101]^ significantly advanced its application. Later, chemical vapor deposition (CVD) techniques were developed, enabling the synthesis of large-area few-layer graphene on Ni foils.^[102]^ Subsequently, the controllable growth of single-crystal monolayer graphene on copper foil has been demonstrated, achieving high uniformity over relatively large areas.^[103]^ More recently, epitaxial graphene (EG) growth on SiC has emerged as another promising approach to produce large-area graphene, with precise control over layer thickness down to a single atomic layer.^[104]^ Unlike CVD-grown graphene, which typically requires transfer to other substrates, EG forms directly on the SiC surface, making it more suitable for device fabrication. Importantly, EG not only retains the key electronic properties of exfoliated graphene^[105,106]^ but also exhibits novel characteristics due to the presence of the SiC substrate—offering potentially valuable insights for biosensing development.

### 3.2. TMDCs

Unlike graphene, which consists of a single layer of carbon atoms, TMDCs have an *MX*_*2*_ structure, i.e., one transition metal layer sandwiched between two chalcogen layers, where the *M* represents the transition metal (e.g., W, Mo, Nb, etc.) and *X* represents the chalcogen (e.g., S, Se, etc.). TMDCs exhibit a wide range of bandgaps and band alignments.^[107,108]^ Notably, they demonstrate an indirect-to-direct bandgap transition when their thickness changes from multilayer to single layer.^[73]^

In biosensing, monolayer TMDCs have garnered increasing interest due to their excellent electrical and optical properties.^[74,109]^ Their semiconducting nature, with a tunable bandgap, has been widely leveraged in the fabrication of FET biosensors.^[110–113]^ Beyond that, the layer-dependent PL properties of TMDCs, their strong fluorescence quenching capabilities, and strain- or doping-induced Raman shifts have advanced their application in label-free optical biosensing.

Among all TMDCs, MoS_2_ has been extensively studied for the detection of various bioanalytes, including immune components, proteins, DNA, and biochemical compounds.^[114]^ Monolayer MoS_2_ exhibits varying Förster resonance energy transfer (FRET) quenching efficiencies toward different dye-labeled biomolecules, which has been widely exploited in the design of biosensors for distinguishing certain biomolecules. More interestingly, MoS_2_ has also demonstrated its potential for label-free detection, based on its PL modulation upon interaction with attached chemical or biological molecules.^[72,115,116]^ For instance, Catalán-Gómez et al.^[72]^ developed a PL biosensor for the breast cancer biomarker miRNA-21 using functionalized monolayer MoS_2_ flakes (Figure 3b). Upon hybridization—i.e., when the target miRNA-21 bound to the immobilized complementary sequence—the PL of MoS_2_ exhibited a redshift, demonstrating both the specificity and viability of the designed biosensor.

Besides MoS_2_, WS_2_ is another widely explored TMDC for biosensing applications.^[35]^ The most stable configuration of WS_2_ is its semiconducting 2H phase, which shares similar structural and electronic characteristics with MoS_2_. Owing to its strong light absorption, integrating WS_2_ into SPR biosensors can significantly enhance sensitivity. The intrinsically larger spin-orbit coupling of WS_2_^[117]^ relative to MoS_2_ leads to stronger optical absorption at shorter wavelengths, which, under certain configurations, enables WS_2_-based SPR biosensors to outperform their MoS_2_ counterparts.^[118]^ Expanding beyond MoS_2_ and WS_2_, emerging TMDCs, such as ReS_2_,^[119]^ MoTe_2_, and WTe_2_^[120]^ have also demonstrated potential in label-free optical biosensing. For instance, Kitadai et al.^[121]^ evaluated the SERS activity of various TMDCs for detecting copper phthalocyanine (CuPc) molecules, including WTe_2_, WSe_2_, SnS_2_, SnSe_2_, TiSe_2_, TiS_2_, and TiTe_2_, and found that several of them—particularly WSe_2_ and SnS_2_—exhibited performance comparable to graphene.

### 3.3. MXenes

MXenes are a rapidly emerging class of 2D transition metal carbides, nitrides, or carbonitrides, typically with an *M*_*n*+*1*_*X*_*n*_*T*_*x*_ structure, where *M* represents the transition metal (e.g., Ti, V, Nb, etc.), *X* represents carbon and/or nitride, and *T*_*x*_ represents the surface functional groups, usually ─OH, ─O, and ─F.^[77]^ The synthesis of MXenes involves selective etching of the *A*-element layer from *MAX* phases, where the formed type of surface terminations is attributed to the etching environment. These surface groups are essential for the stability, dispersibility, and biosensing functionality of the synthesized MXenes. Additionally, they can also be modified to tailor the electrical, optical, and chemical properties of the material.^[76]^

The quenching capability of MXenes make them highly attractive for developing fluorescent sensors.^[122,123]^ For example, Xie et al. employed Ti_3_C_2_T_x_ MXene nanosheets for real-time detection of Ag^+^ ions in aqueous solution, achieving a low LOD of 0.035 μM with high selectivity.^[123]^ Notably, 2D MXenes are hydrophilic due to their surface groups and exhibit excellent metallic conductivity, both of which are favorable for label-free optical biosensing.^[76, 124–127]^ Their tunable optical properties, i.e., plasmonic behavior in the near-IR (NIR) to mid-IR (MIR) regime, enable enhanced light–matter interactions and can be exploited in SPR biosensors.^[77]^ For example, Gan et al.^[128]^ developed a Ti_2_C MXene-based SPR biosensor for detecting heavy metals with high sensitivity. Their study showed that the presence of an MXene overlayer significantly enhanced SPR sensor performance. Moreover, the abundant functional groups on MXene surfaces enable efficient immobilization of bioanalytes and facilitate strong charge transfer between MXenes and the analytes, making MXenes a promising substrate for SERS detection.^[126,127]^ For instance, as demonstrated in Figure 3c, Li et al.^[100]^ developed a uniform 2D Ti_3_C_2_T_x_/Ag NPs SERS substrate exhibiting an enhancement factor (EF) of 3 × 10^8^. Attributed to synergistic CM and EM mechanisms, this SERS platform enabled R6G detection down to the fm level. Furthermore, MXenes are highly biocompatible and solution-processable, making them suitable for integration into various label-free optical biosensing platforms.^[76,124,125]^

### 3.4. BP

BP is one of the most stable phosphorus allotropes,^[129]^ compared to white and red phosphorus. It is a layered 2DM with phosphorus atoms arranged in a puckered honeycomb structure. Owing to its unique physicochemical properties—such as high carrier mobility, strong photothermal and photoconductive responses, and a layer-dependent direct bandgap—BP has been explored for a wide range of applications, including batteries,^[130]^ supercapacitors,^[131]^ optoelectronics,^[132]^ and biosensing.^[82]^

BP nanosheets have been utilized in the development of fluorescent biosensors^[133,134]^ due to their strong fluorescence quenching capability. Unlike graphene, BP exhibits high in-plane anisotropy in both its optical and electrical properties, which is advantageous for label-free optical biosensing. This anisotropy enables polarization-sensitive detection, tunable light–matter interactions, and the design of highly selective photonic and optoelectronic biosensors. For instance, Lin et al.^[81]^ studied the anisotropic charge transfer between few-layer BP and CuPc molecules, which induced polarization-dependent Raman signal enhancement, as shown in Figure 3d. These findings shed light on the electronic properties of anisotropic BP 2D sheets and their applications in biosensing. Moreover, BP has broadband optical absorption in the visible to NIR regime,^[83]^ making it favorable for IR-based optical biosensing. Its rich surface chemistry and high biocompatibility further facilitate strong interfacial interactions with biomolecules, enabling label-free detection mechanisms based on photo-induced charge transfer^[135–137]^ and dielectric modulation.^[138]^

Recent studies have demonstrated the potential of BP nanosheets as a promising substrate for SERS-based biosensors. For instance, Li et al.^[86]^ developed a SERS substrate that integrated BP nanosheets with noble metal NPs (Au and Ag NPs), for the detection of fungicides, demonstrating excellent sensitivity and reproducibility. Liu et al.^[84]^ developed BP-based nanohybrids as multifunctional theranostic platforms for drug delivery, phototherapy, and bioimaging, revealing extraordinary NIR photothermal transduction efficiency and drug delivery capacity, as well as demonstrating good SERS activity under NIR excitation. The minimal spectral interference makes BP nanosheets highly desirable for the SERS detection of biological samples.

However, the practical application of BP is constrained by its instability under ambient conditions, as it degrades upon exposure to air and humidity. To preserve its properties for long-term biosensing, two major strategies are commonly adopted: i) encapsulation, where a protective layer is coated onto BP; or ii) surface modification, i.e., creating a passive layer to prevent oxidation.^[134]^

### 3.5. hBN

Known as “white graphene,” hBN has a similar honeycomb lattice structure but is composed of alternating boron and nitrogen atoms instead.^[37,46]^ Unlike graphene, hBN is electrically insulating with a large bandgap, making it optically transparent across a broad spectral range from ultraviolet (UV) to the NIR.^[25]^ This makes hBN an ideal substrate and support layer for biosensing applications where minimizing background interference is crucial. Though hBN is not typically used as the direct transducer, its unique optical and surface properties make it indispensable in advancing label-free optical biosensors.^[20]^ Because of its surface smoothness, excellent thermal stability, and chemical inertness, hBN can act as a protective layer or dielectric spacer integrated into label-free optical biosensors, particularly when the transducer material is unstable or prone to oxidation, such as TMDCs. For example, Kim et al.^[87]^ used hBN as an insulating layer to protect metal NPs from photothermal and oxidative damage during SERS detection, thereby improving the overall stability of the system. Additionally, stronger EM fields were generated at the narrow gaps on the hBN surface—referred to as “hot spots” in Figure 3e—which enhanced the Raman scattering signal of R6G when the molecules were in close proximity to these regions.

More recently, scientists have demonstrated label-free optical biosensing using hBN through imaging-based approaches. For instance, Zhang et al.^[139]^ used the selectively activated defect emissions of the hBN surface to achieve label-free single-molecule detection and identification of biomolecules. By using spectrally resolved super-resolution microscopy together with ML, they were able to identify five distinct amino acids. In another work, Sülzle et al.^[140]^ investigated the interactions between DNA and the hBN surface through direct imaging of variations in local optical scattering, where the signal was higher at engineered defects than in pristine regions. These findings highlight hBN as a versatile platform for single-molecule biosensing and pointed out its value and potential in advancing hybrid label-free optical biosensing systems.

### 3.6. Antimonene

Recently, other monoelemental 2DMs (Xenes) beyond graphene and phosphorene, such as arsenene, antimonene, and silicene, have emerged as promising materials for biosensing applications.^[92,141]^ Owing to their unique electronic properties, large surface area, ultrathin nature, and high flexibility, Xenes have been widely explored in advancing FET biosensors,^[141]^ offering ultrahigh sensitivity in detecting proteins, DNA/RNA, and small molecules. Among these Xenes, antimonene has recently demonstrated its promise in developing label-free optical biosensors,^[71]^ such as SPR biosensors for the ultrasensitive detection of miRNA.^[88]^ Antimonene shares the same *sp*^*2*^ honeycomb lattice as graphene but possesses superior electrochemical properties compared to graphene and other 2DMs.^[89,90]^ It has high carrier mobility, a layer-dependent tunable bandgap, and exhibits strong spin-orbit coupling.^[91]^ In particular, its high work function, large adsorption energy, high stability, and hydrophilicity make it highly preferable as a biomolecular recognition layer in constructing SPR biosensors.^[71,92]^ In 2019, Xue et al.^[88]^ reported that antimonene had a substantially stronger interaction with ssDNA than graphene, based on first-principles density functional theory (DFT) calculations. Motivated by this insight, they developed an SPR sensor for miRNA detection (Figure 3f) and achieved an attomolar-level detection limit. Since then, a growing number of studies^[142–147]^ have utilized antimonene in layered SPR biosensors to further improve sensitivity, highlighting its potential in the development of biosensing platforms with high performance.

## 4. Mixed-Dimensional Hybrid Nanostructures for Label-Free Optical Biosensing

In the previous section, we briefly introduced and discussed the properties of different 2DMs and their applications in label-free optical biosensing. With the trend in biosensor design moving toward more controllable, functionally tailored, and high-performance platforms with improved stability, there has been growing interest in designing biosensors by combining 2DMs to create hybrid materials with enhanced properties and functionalities. In this section, we provide an overview of mixed-dimensional hybrid nanostructures composed of 2DMs and other nanomaterials, along with their examples in label-free optical biosensing.

### 4.1. Hybrid Nanostructures and Examples in Label-Free Optical Biosensing

**Figure 4.**
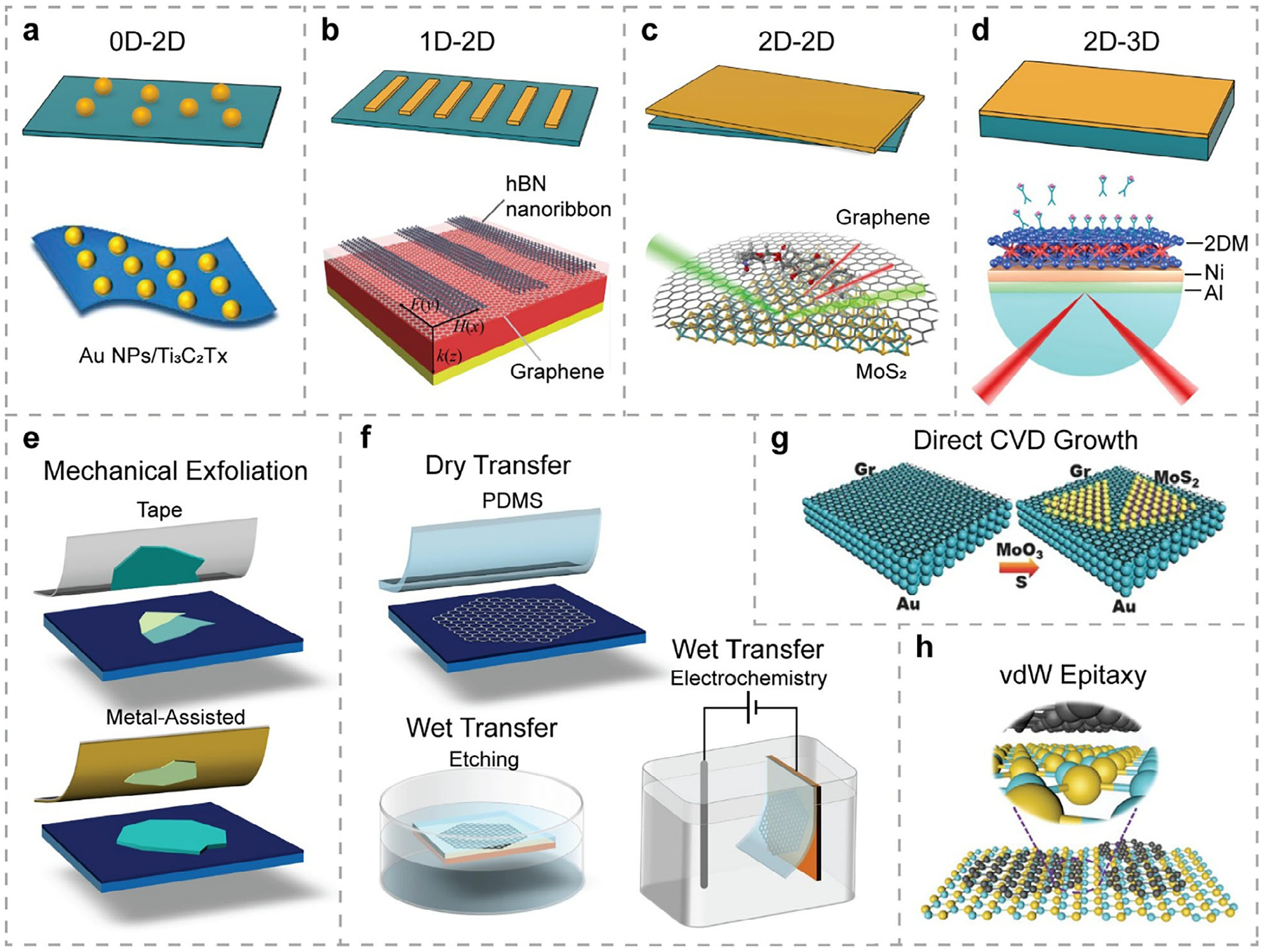
Mixed-dimensional hybrid nanostructures and typical fabrication techniques of 2D vdWHs. a) Schematic of a 0D-2D hybrid structure and example of Au NPs/MXene used as SERS substrate for bacterial detection. Reproduced with permission.^[153]^ Copyright 2021, Elsevier. b) Schematic of a 1D-2D hybrid structure and example of theoretically proposed hBN nanoribbons/graphene hybrids for SPR biosensing. Reproduced with permission.^[154]^ Copyright 2020, IOP Publishing. c) Schematic of a 2D vdWH and example of graphene/MoS_2_ heterostructure for label-free optical biosensing. Reproduced with permission.^[63]^ Copyright 2022, American Chemical Society. d) Schematic of a 2D-3D hybrid structure and example of integrated 2DMs for enhanced SPR performance. Reproduced with permission.^[155]^ Copyright 2023, MDPI. e) Mechanical exfoliation. f) Dry and wet transfer techniques. g) Direct CVD growth of MoS_2_ on graphene. Reproduced with permission.^[156]^ Copyright 2015, Wiley. h) Schematic of vdW epitaxy. Reproduced with permission.^[157]^ Copyright 2022, Wiley.

To enhance the performance and versatility of label-free optical biosensors, recent efforts have focused on engineering hybrid nanostructures by integrating different 2DMs. The physically combined 2DMs are known as 2D vdWHs,^[28,148]^ where the assembly is attributed to weak vdW forces rather than chemical bonds. The vdWHs can be vertically stacked—such as graphene/MoS_2_,^[63]^ hBN/graphene,^[149]^ or WSe_2_/graphene heterostructures^[150]^—or laterally stitched, such as WS_2_/MoS_2_^[151]^ or WSe_2_/MoSe_2_ heterostructures.^[152]^ In this review, we focus on vertically stacked heterostructures, as schematically illustrated in Figure 4c. Since these vertical vdWHs are composed of 2DMs, they inherit the properties of each individual 2DM and have been extensively explored for biosensing applications. For instance, in label-free optical biosensing, a vertically stacked graphene/MoS_2_ heterostructure (shown in Figure 4c) was reported^[63]^ in which both the PL response of MoS_2_ and the GERS signal from graphene were used to detect doxorubicin (DOX), a cancer drug, with high sensitivity and selectivity. Additionally, because the constituent 2DMs possess complementary properties, their vdWHs can exhibit unique or improved electronic, optical, and mechanical characteristics compared to their individual components, enabling novel physical phenomena and advancing various application fields,^[29,30]^ including label-free optical biosensing. Moreover, vdWHs offer additional tunability of their overall electrical and optical properties via chemical doping, electrostatic gating, nanostructure design, and control over stacking configurations (layer arrangement, twist angles, formation of Moiré patterns, etc.).^[29]^ Importantly, vdWHs can mitigate the disadvantages of unstable 2DMs, such as degradation and oxidation under ambient conditions, by using stable 2DMs as protective overlayers. For example, TMDCs are known to degrade upon exposure to air. By transferring a graphene layer on top of MoS_2_, the overall stability of the biosensor can be significantly improved^[63]^ (Figure 4c).

Inspired by the concept of 2D vdWHs integration, researchers have expanded the design beyond purely 2D to mixed-dimensional hybrid nanostructures—such as 0D–2D, 1D–2D, and 2D–3D systems—which also may not rely solely on vdW interactions. Instead, these structures often integrate multiple material classes using a range of physical or chemical strategies to realize complementary or enhanced biosensing functions. For instance, noble metal NPs, such as Au and Ag, exhibit excellent plasmonic properties and localized surface plasmon resonance (LSPR), which are advantageous for SPR and SERS biosensing. When combined with 2DMs—materials that have large surface areas to support functionalization and effective immobilization of bioanalytes—0D–2D hybrid nanostructures can substantially enhance the overall performance of label-free optical biosensors. Indeed, researchers have actively explored various combinations of metallic and non-metallic NPs, as well as complex compositions, with 2DMs to improve SERS-based biosensors.^[46,47]^ For example, Yu et al.^[153]^ developed an Au NPs/Ti_3_C_2_T_x_ hybrid substrate (Figure 4a) for SERS detection of bacteria, achieving high sensitivity, uniformity, and stability.

In SPR biosensors, metallic films are essential for supporting surface plasmon polaritons, which enable label-free detection. However, these films are often prone to oxidation and corrosion. Using 2DMs as protective overlayers on metallic films improves the stability of SPR biosensors. In addition, the large surface area of 2DMs facilitates efficient receptor functionalization, and their high optical absorbance can further enhance SPR sensor performance. Furthermore, the tunability of 2DMs—by adjusting the number of layers, doping levels, and surface functionalization—provides additional flexibility in tailoring SPR sensor designs to meet specific detection requirements. As a result, 2D-3D integration, illustrated in Figure 4d—i.e., hybrid structures of 2DMs with metallic films, or multicomponent architectures comprising alternating 2DMs and metallic layers—has become a widely adopted approach in SPR biosensor development. For example, Hu et al.^[155]^ systematically studied the influence of various 2DMs on Ni/Al bimetallic SPR biosensors, finding that both the real and imaginary components of the 2DMs’ RI significantly affected sensor sensitivity. By optimizing material combinations, stacking order, and layer thickness, a maximum angular sensitivity of 542 deg RIU^−1^ was achieved using a BP (1L)/Ni (22 nm)/Al (40 nm) hybrid configuration.

Hybrids of 1D nanostructures and 2DMs represent another promising configuration—integrating NWs, nanoribbons, or nanobelts with 2DMs, as illustrated in Figure 4b—that can benefit both SPR- and SERS-based biosensors. For example, a 1D-2D hybrid structure of hBN nanoribbons/graphene^[154]^ was proposed to enhance SPR performance.

These hybrid nanostructure designs, which extend beyond 2D–2D systems, open new pathways for advancing label-free optical biosensing. However, to fully exploit their potential, a deeper understanding of the underlying light-matter interactions^[29,30]^ and sensing mechanisms is required. Additionally, synthesis methods could significantly influence the morphology, surface chemistry, and uniformity of these materials, thereby affecting their performance in biosensing applications. Depending on the hybrid configuration and materials chosen, appropriate fabrication strategies must be carefully selected to ensure the desired functionality. In the following subsection, we briefly discuss fabrication strategies for 2DMs and their hybrid nanostructures.

### 4.2. Synthesis and Fabrication Techniques

Various methods and techniques have been developed to produce 2DMs and their 2D vdWHs, including mechanical exfoliation^[158]^ (Figure 4e), dry and wet transfer techniques^[159]^ (Figure 4f), liquid-based methods, CVD (Figure 4g), and direct epitaxial growth^[157]^ (Figure 4h). Mechanical exfoliation using Scotch tape was the first reported method for isolating monolayer graphene in 2004,^[101]^ and has since been widely used for exfoliating other 2DMs from their bulk counterparts. This technique involves repeatedly picking up and stamping layered materials with Scotch tape or a polymer stamp, as illustrated the upper panel of Figure 4e, which can yield single-layer flakes over relatively large areas. The resulting 2DMs are fairly clean and smooth, with fewer defects. However, the size, shape, and uniformity of layer numbers are difficult to control. Besides, the time-consuming and manual process makes it unsuitable for scalable production. Recently, metal-assisted mechanical exfoliation has been introduced (as shown in the lower panel of Figure 4e), where a metal film is directly evaporated onto the bulk crystal surface.^[160,161]^ It was reported that the thickness and type of metal film can affect the number of layers exfoliated.^[161]^ However, to expose the 2DM surface for 2D vdWH assembly, the metal layer must be removed by chemical etching, which often introduces extra contamination or doping to the exfoliated 2D flakes.^[161]^

Liquid-based methods, such as hydrothermal and solvothermal synthesis, as well as liquid-phase exfoliation, offer cost-effective and scalable routes for producing 2D nanosheets. For example, ultrasonic-assisted liquid-phase exfoliation has been successfully applied to synthesize BP nanosheets^[84,136,137]^ and Ti_3_C_2_T_x_ Mxenes.^[153, 162–164]^ The 2D sheets synthesized by these methods often have functionalized surface groups. However, the crystallinity and interface sharpness are often not as high-quality as those produced by CVD or other methods.

CVD is one of the most widely used techniques for the controlled, wafer-scale synthesis of high-quality, crystalline 2DMs. For example, CVD has been used to grow monolayer graphene on copper,^[165]^ MoS_2_ on Si/SiO_2_,^[63]^ and hBN on sapphire.^[166]^ To fabricate 2D vdWHs from these CVD-grown 2DMs, transfer techniques—either wet or dry—are often required. Wet transfer methods (as illustrated in the lower two panels of Figure 4f) typically involve either chemical etching of the growth substrate or bubbling from electrochemistry to lift the as-grown 2DMs off the substrate. In both cases, a support layer, e.g., spin-coated polymethyl methacrylate (PMMA) or drop-cast polycarbonate, is used to protect the 2DM during the transfer process. While wet transfer can yield large-area, uniform monolayers, it can introduce hard-to-remove chemical residues and, consequently, affect the optical and electrical properties of the resulting vdWHs. Additionally, post-treatments are often necessary for wet transfer methods, such as heating the substrate with transferred 2DMs for better adhesion, removal of the polymer support layer, and annealing to relax the 2DMs and reduce wrinkles. For dry transfer, on the other hand, a polydimethylsiloxane (PDMS) stamp is used to directly peel off the 2DMs and transfer them to a new substrate without any post-treatment process (as shown in the upper panel of Figure 4f).

For applications requiring precise control over the interface and stacking order, direct growth of one 2DM over another grown or transferred 2DM is a straightforward approach to fabricate vdWHs. For example, researchers have used controlled CVD to directly grow MoS_2_^[156]^ and WSe_2_^[150]^ on top of graphene. In other works, CVD-based epitaxial growth was successfully achieved for MoS_2_^[167]^ and WSe_2_^[168]^ on mechanically exfoliated hBN. Epitaxial growth between TMDC crystals was also explored, such as growing MoTe_2_ monolayer on MoS_2_ substrate.^[169]^ Need to mention that, vdW epitaxy can be repeated multiple times to fabricate complex multicomponent heterostructures. For example, Lin et al.^[170]^ used trilayer EG as a substrate to directly grow WSe_2_ by a combination of oxide powder vaporization and metal-organic CVD (MOCVD) process. Subsequently, a second layer of MoS_2_ was grown on top of the initially grown heterostructure by vaporization of MoO_3_ and sulfur, forming MoS_2_/WSe_2_/EG vertical heterostructures.

2D–3D hybrid nanostructures can be fabricated using all of the aforementioned methods. For 0D–2D systems, i.e., NPs/2DM hybrids, the synthesis methods are slightly different. A commonly used approach is chemical reduction, where metal precursors and nanosheets are mixed and reduced in situ using suitable agents, as demonstrated in the synthesis of Au NPs/MXene^[153,171]^ hybrid structures. In other studies, scientists have also used self-assembly between nanosheets and metal NPs,^[100,162,172]^ driven by electrostatic adsorption. This process often involves ultrasonication or stirring to ensure even dispersion of the NPs and to prevent aggregation. To directly deposit NPs onto 2DMs on a substrate, physical vapor deposition (PVD) methods—such as sputtering and electron beam evaporation—are commonly used. For example, Mei et al.^[173]^ used magnetron sputtering and electron beam evaporation to deposit Ag and Au layers, respectively, in the fabrication of Au/graphene/Ag/ZnO hybrid nanostructures. Lu et al.^[174]^ used magnetron sputtering to deposit an Ag layer onto a 3D polystyrene (PS) substrate for fabricating graphene oxide (GO)/Ag/3D PS hybrid nanostructures.

The fabrication of 1D–2D hybrids often requires additional patterning techniques, such as electron-beam lithography or reactive ion etching, beyond the fabrication of 2D vdWHs. For example, graphene nanoribbons have been fabricated on the Si/SiO_2_ substrate using reactive ion etching.^[57]^ In another study, patterned graphene nanoribbons were created on the CaF_2_/SiO_2_/Si substrate using electron-beam lithography.^[175]^

## 5. Recent Advances in 2DMs and Their Hybrid Nanostructures for Label-Free Optical Biosensing

2DMs and their mixed-dimensional hybrid nanostructures have emerged as promising platforms for the development of label-free optical biosensors. In this section, we provide an overview of recent advances in their design and application for SERS- and SPR-based biosensing. Furthermore, we highlight the development of multifunctional sensing platforms that enable multiplexed and multimodal detection, along with the incorporation of ML techniques to enhance signal interpretation and overall sensor performance.

### 5.1. Recent Advances in SERS-Based Biosensing

Conventional SERS substrates are predominantly based on noble metal NPs, i.e., Au NPs, Ag NPs, etc. However, because of their proneness to aggregation, poor stability, limited reproducibility, and the high cost of noble metal precursors, scientists have explored alternative materials beyond pure metallic NPs as SERS substrates. 2DMs, owing to their unique and tunable optical and electrical properties, large surface-to-volume ratios, and high biocompatibility, have attracted tremendous attention for the development of promising SERS substrates. In this subsection, we discuss recent innovations in leveraging novel 2DMs and their mixed-dimensional hybrid nanostructures to advance SERS-based biosensing toward higher sensitivity and practical feasibility.

#### 5.1.1. Pure 2DMs

To date, several 2DMs have demonstrated strong potential as SERS substrates. Graphene, the most widely studied 2DM, has been extensively employed in GERS and biosensing applications.^[51]^ TMDCs, such as MoS_2_ and WS_2_, have also demonstrated their capability in enhancing Raman signals. More recently, other TMDCs^[176–180]^ have emerged as promising SERS-active materials. For example, Ekoya et al.^[176]^ demonstrated the SERS detection of dye molecules—R6G and Nile blue A (NBA)—using 2H-TaS_2_ as the substrate, where LODs of 3.01 × 10^−18^ m and 4.05 × 10^−21^ m were achieved for R6G and NBA, respectively. The maximum EF for R6G reached 1.3 × 10^14^, owing to the strong interactions between the dye molecules and the 2H-TaS_2_ substrate. Li et al.^[177]^ utilized 2D HfTe_2_ nanosheets as a SERS substrate for R6G detection, where an EF of 2.32 × 10^6^ was achieved. Notably, this SERS platform was further explored to detect uric acid, a gout disease-associated biomarker, where a LOD of 0.1 mmol L^−1^ was obtained, illustrating its potential for nano-diagnostics. Since early diagnosis is essential for timely interventions, it is of great interest to detect disease biomarkers. In another study, few-layer semi-metallic MoTe_2_ was used as a SERS substrate for detecting *β*-sitosterol,^[178]^ a lipid disease biomarker, achieving a LOD of 1 nm. The Raman signal enhancement was attributed to the charge-transfer process upon the formation of surface-analyte complex. Compared with noble-metal SERS substrates, the MoTe_2_ film exhibited higher homogeneity and reproducibility, as well as reusability.

**Figure 5.**
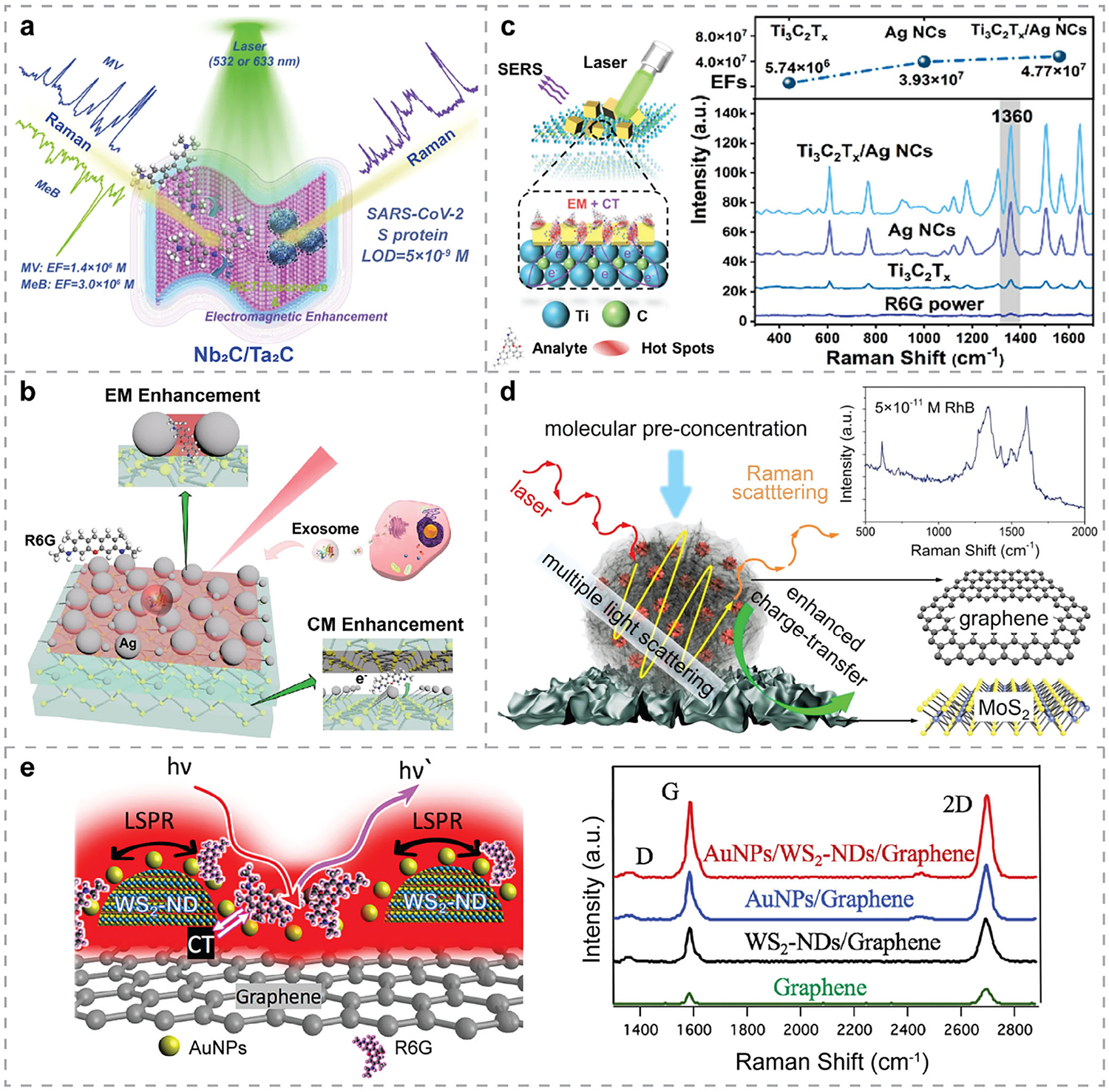
Recent advances in using 2DMs and 0D-2D hybrid nanostructures for SERS biosensing. a) MXene-based SERS platform for highly sensitive SARS-CoV-2 S protein detection enabled by the synergistic effects of charge-transfer resonance and EM enhancement. Reproduced with permission.^[187]^ Copyright 2021, Springer Nature. b) Single-molecule SERS detection using Ag NPs/BP hybrid nanosheets. Reproduced with permission.^[135]^ Copyright 2022, Springer Nature. c) Ag NCs/Ti_3_C_2_T_x_ SERS substrate for therapeutic drug monitoring facilitated by the synergistic CM and EM effects. Reproduced with permission.^[189]^ Copyright 2023, Elsevier. d) Hybrid nanostructures of Gr MFs/wrinkled MoS_2_ used as SERS substrate for ultrasensitive molecular sensing. Reproduced with permission.^[190]^ Copyright 2020, Elsevier. e) Au NPs/WS_2_ NDs/graphene hybrid nanostructures with enhanced SERS performance. Reproduced with permission.^[191]^ Copyright 2020, American Chemical Society.

Beyond graphene and TMDCs, MXenes have also emerged as compelling SERS substrates. Among them, Ti_3_C_2_^[78,163,181,182]^ and Ti_2_N^[183]^ are the most widely used. More recently, a broader range of MXenes^[184–187]^ has shown strong potential for SERS-based biosensing. For instance, 2D V_2_C and V_4_C_3_^[185]^ achieved R6G detection with a LOD of 10^−7^ m, where a layer-dependent EF was observed. The SERS performance was further enhanced by a 2D downsizing strategy and molecular enrichment, reaching a LOD of 5 × 10^−9^ m. In another study, He et al.^[186]^ synthesized TiVC through a single-step chemical etching process and evaluated its SERS performance, achieving a femtomolar-level LOD for R6G with an EF of 10^12^. Similarly, Lan et al.^[188]^ assessed the SERS performance of Ta_4_C_3_ and Nb_4_C_3_ for R6G and CV detection. Owing to the photo-induced charge transfer between MXene and adsorbed molecules, an EF of ~10^5^ and a LOD of 10^−7^ m were achieved. These studies pave the way for using MXenes as promising SERS substrates; however, more research needs to be carried out for practical biosensing applications. Impressively, Peng et al.^[187]^ theoretically designed and experimentally studied the excellent SERS performance of Nb_2_C and Ta_2_C, with the latter showing remarkable capability in detecting the SARS-CoV-2 S protein (Figure 5a). An exceptionally low LOD of 5 × 10^−9^ m was reported, indicating the feasibility of real-time monitoring and early detection of novel coronavirus, and highlighting MXenes as a strong candidate for practical SERS applications.

hBN has also been investigated as the SERS substrate. Both exfoliated^[75]^ and CVD-grown^[192]^ hBN have shown enhanced Raman signals of probe molecules, with the enhancement attributed primarily to dipole-dipole interactions and remaining unaffected by the number of hBN layers. However, due to its relatively low EF, hBN is unlikely to be used in practical applications without additional design or engineering. Similarly, BP sheets also exhibit limited enhancement. Even with special engineering, such as creating nano-void arrays on BP sheets,^[193]^ which consequently enhanced the local electric field for SERS detection, the EF of R6G remains significantly lower than those achieved with other 2DM-based SERS substrates. Table 2 summarizes recently emerged 2DMs employed as SERS substrates.

#### 5.1.2. Hybrid Nanostructures Used as SERS Substrates

Different from SERS substrates composed of plasmonic NPs, where enhancement is dominated by the EM mechanism, most 2DM-based SERS relies on the CM. While 2DM-based SERS substrates offer distinct advantages over noble metal NP-based counterparts, such as high homogeneity and reproducibility, their EFs and sensitivities remain comparatively lower. To overcome these limitations, the design of 0D-2D hybrid nanostructures—integrating metallic NPs with 2DMs—has emerged as a promising strategy to achieve significantly enhanced SERS performance through synergistic CM and EM effects, while simultaneously improving stability, reproducibility, selectivity, and biocompatibility.

The weak EF of hBN has led to its integration with metal NPs for SERS-based biosensing.^[87,192,195,196]^ Similarly, recent studies have also demonstrated the SERS capability of metal NPs/BP hybrid nanostructures^[84,86,135,197]^ and their potential for highly sensitive biomarker detection. For instance, Kundu et al.^[197]^ fabricated Ag NPs/BP hybrids as the SERS substrate for the sensitive detection of sepsis biomarkers. The Raman spectra of the biomarkers were sharp and significantly enhanced in the presence of Ag NPs/BP flakes, with no background interference from BP itself. The EF reached the order of 10^14^, and the LODs for both prognostic and diagnostic biomarkers were as low as 1000 fm and 100 fm, respectively. Moreover, this high-performance SERS platform revealed fingerprint peaks of sepsis biomarkers at low frequencies, paving the way for future development of multiplexed detection of these biomarkers in more complex biological matrices. In another work, Lin et al.^[135]^ developed Ag NPs/BP hybrid nanosheets that exhibited remarkable SERS performance and single-molecule detection capability, as illustrated in Figure 5b. Owing to the synergistic resonance enhancement of molecular resonance, photo-induced charge transfer, and the EM effect, this SERS substrate achieved a low LOD of 10^−20^ mol L^−1^ for R6G. Utilizing a new polarization-mapping method combined with ML, the Ag NPs/BP hybrid nanosheets enabled detection and recognition of different tumor exosomes at the single-molecule level, demonstrating their potential for biomonitoring and early cancer detection. Beyond the single function of SERS sensing, Ma et al.^[198]^ fabricated a hybrid nanostructure of Au NPs and Ag NPs on BP nanosheets for both detection and degradation of hazardous materials. The abundant active “hot spots” induced by the Au-Ag NPs, along with the charge transfer between BP and probing molecules, led to a low LOD of 4.5 × 10^−10^ m for 4-Mercaptobenzoic acid (4-MBA). For practical application, this SERS substrate was further incorporated into flexible SERS chips for in situ monitoring of thiram, where a LOD of 2.6 × 10^−6^ mg mL^−1^ was achieved. Additionally, owing to the formation of a metal–semiconductor heterojunction, this SERS chip possesses photocatalytic self-cleaning properties, enabling repeatable and reliable assays.

Hybrid structures of metal NPs and MXenes^[100, 153, 162, 171, 172, 199–202]^ have also demonstrated high SERS performance and, especially, their potential in practical biosensing applications related to food safety^[172,202,203]^ and human health.^[100,153,162,200,201]^ For instance, Au NPs/TiC nanosheets were developed and employed for the SERS detection of chlorpromazine,^[199]^ achieving a wide linear range (10^−1^–10^−10^ m) and an ultralow LOD of 3.92 × 10^−11^ m. In another example, Ag NPs/Ta_4_C_3_ hybrids were fabricated and explored as SERS substrates for the sensitive detection of pesticide ziram,^[202]^ achieving a LOD of 10^−6^ m. Liu et al.^[162]^ fabricated Ag NPs/Ti_3_C_2_ hybrid nanosheets for the sensitive detection of 4-MBA molecules, exhibiting excellent SERS performance, long-term stability, and good uniformity. MXene-based nanohybrids have also been employed to develop SERS chips that combine superior sensing capability with additional practical features. For example, a wearable SERS sensor based on Au NPs/TiVC hybrids was engineered for physiological monitoring.^[201]^ In addition to the sensitive detection of nicotine, methotrexate, nikethamide, and 6-acetylmorphine in sweat, this sensor also exhibited good mechanical durability and long-term stability. In another example, Au NPs/Ti_3_C_2_ hybrids, synthesized via electrostatic self-assembly, were not only capable of sensitive SERS detection of *E. coli* and *B. subtilis*,^[153]^ but also exhibited antibacterial and photothermal sterilization effects. Chen et al.^[200]^ fabricated a flexible SERS chip by integrating Ag NPs/Ti_3_C_2_T_x_ hybrids with PMMA for SARS-CoV-2 detection. The synergistic effects from Ag NPs and MXene led to high SERS performance, whereas PMMA integration provided the sensor chip with both mechanical flexibility and self-rectification functionality. In another study, Au nanostars/Ta_4_C_3_^[204]^ hybrids were used as SERS substrates and exhibited a LOD of 10^−9^ m for detecting 4-aminothiophenol (PATP). By combining with filter paper, the fabricated paper-based SERS substrate was able to detect thiram residues after directly wiping the apple surface. Notably, Xue et al.^[189]^ developed a SERS substrate based on Ag nanocubes (NCs)/Ti_3_C_2_T_x_ MXene hybrid nanostructures (Figure 5c) for therapeutic drug monitoring. From the Raman spectra shown in Figure 5c, the maximum enhancement was achieved when integrating Ti_3_C_2_T_x_ with Ag NCs, compared to using Ag NCs or Ti_3_C_2_T_x_ alone as the SERS substrate. This superior performance was attributed to dual EM-CM enhancement—specifically, the dual effect of EM “hotspots” formed in the nanoscale gaps between clustered Ag NCs, and effective photo-induced charge transfer between the 2D Ti_3_C_2_T_x_ matrix and target molecules.

In addition to MXenes, MoS_2_ nanosheets—the most common TMDC—have been combined with Au NPs into hybrid structures for SERS-based biosensing.^[205,206]^ With the EM effect of Au NPs and special surface modifications, such a biosensor was able to detect 2,4,6-trinitrotoluene (TNT) explosives^[205]^ with a LOD of 2 × 10^−7^ m and high selectivity. Dou et al.^[206]^ also demonstrated the SERS performance of Au NPs/MoS_2_ for the rapid and sensitive detection of trace malachite green (MG) in flowing water, with a LOD of 1 × 10^−8^ mol L^−1^ and high selectivity. While metal NPs/TMDC hybrid structures have shown promise for SERS biosensing, the fabrication process of metallic nanostructures may cause damage to the TMDC, potentially compromising sensor performance. To address this, Zhang et al.^[207]^ developed a general approach for fabricating such high-performance hybrid platforms. The as-prepared SERS substrates—Au NPs/ReSe_2_, Au NPs/MoS_2_, and Au NPs/PdSe_2_—exhibited LODs of 10^−10^ m, 10^−10^ m, and 10^−12^ m for R6G, respectively.

0D-2D hybrids between metal NPs and graphene or its derivatives—such as GO and reduced GO (rGO)—have also been widely explored as SERS substrates. Example integrations include: Au NPs/graphene,^[208]^ Ag NPs/graphene,^[209–211]^ Au NPs/GO,^[212,213]^ Ag NPs/rGO;^[214]^ as well as hybrids with non-spherical NPs, such as Au nanostars/rGO,^[215]^ Ag NCs/graphene,^[216]^ and triangular Ag nanoplates/GO.^[217]^ These hybrid systems have demonstrated practical and sensitive SERS detection across a broad range of analytes, including pesticide residues,^[218,219]^ Alzheimer’s disease (AD) biomarkers,^[212]^ persistent organic pollutants (POPs),^[216]^ bacteria,^[217]^ and biomolecules associated with early-stage cancer.^[220]^ For instance, Zhao et al.^[221]^ fabricated an ultrasensitive SERS substrate by integrating both Au nanostars and Au@Ag NPs onto GO nanosheets. This platform exhibited analyte enrichment capability and leveraged a multidimensional plasmonic coupling effect induced by the Au@Ag NPs, achieving an ultralow LOD of 10^−11^ m for bilirubin (BR) detection. In another study, Atta et al.^[219]^ developed a GO-coated Au@Ag nanostar-based SERS sensor for pesticide detection, demonstrating ultrahigh sensitivity with LODs of 10, 50, 100, and 100 pm for ziram, phorate, triazophos, and azinphos-methyl, respectively. These findings highlight the strong potential of such hybrid platforms for multiplexed and ultrasensitive biosensing in complex samples. Table 3 summarizes recently reported hybrid structures of noble metal NPs with 2DMs for SERS sensing.

**Figure 6.**
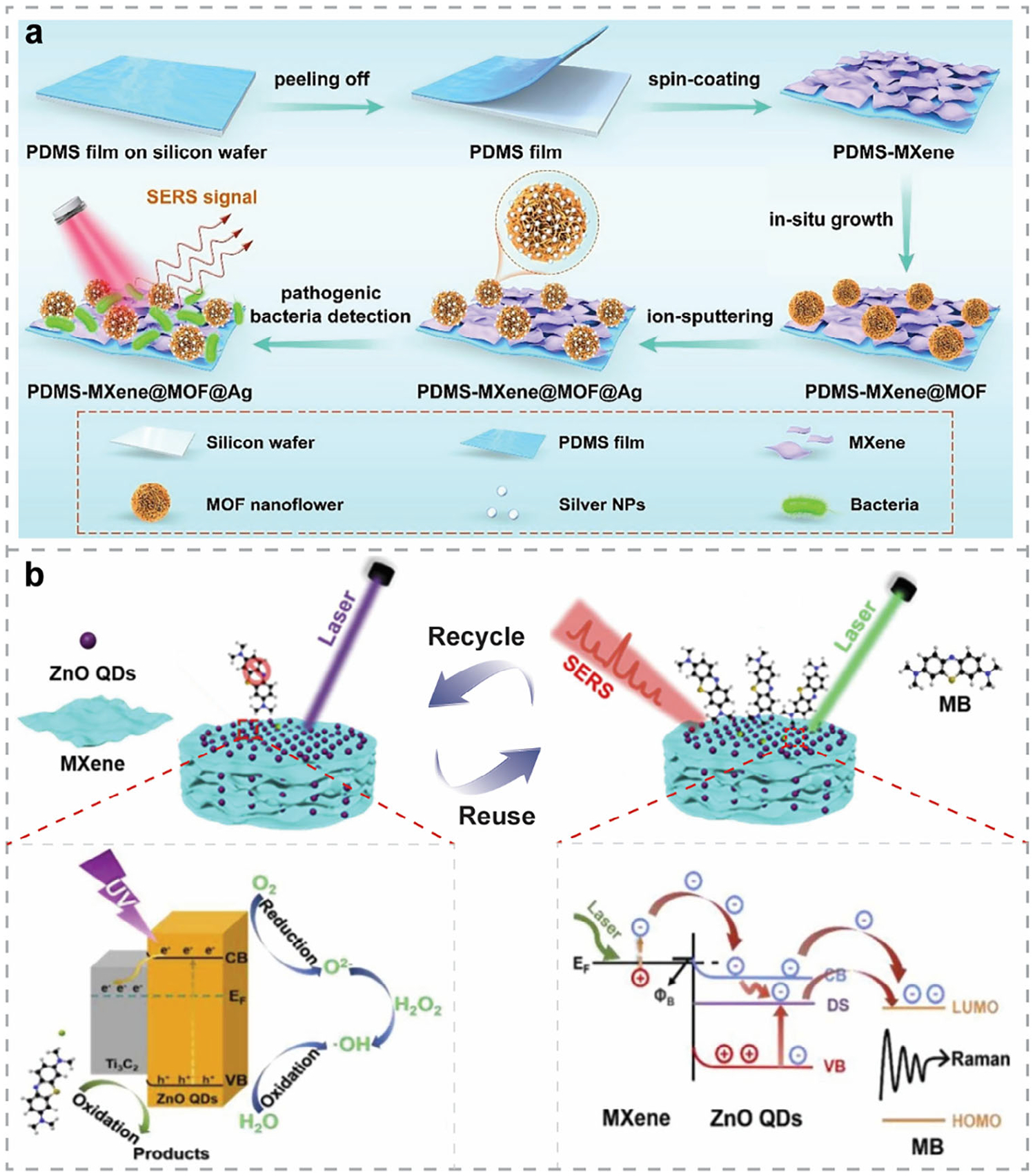
Recent advances in using novel 0D-2D hybrid nanostructures for SERS biosensing. a) Fabricated flexible MOF@Ag/MXene hybrids on PDMS for sensitive SERS detection of pathogenic bacteria. Reproduced with permission.^[231]^ Copyright 2025, Elsevier. b) Recyclable SERS substrate based on ZnO QDs/MXene hybrid structures. Reproduced with permission.^[232]^ Copyright 2024, Elsevier.

Beyond metal NPs, 2DMs have also been integrated with more complex^[164,190,191,222,223]^ or specially engineered^[224–228]^ nanostructures to advance SERS-based biosensing. For example, Qiu et al. designed a heterostructure composed of graphene microflowers (Gr MFs) and wrinkled 2H-phase MoS_2_^[190]^ for plasmon-free SERS detection (Figure 5d). The Gr MFs/wrinkled MoS_2_ hybrids exhibited a LOD of 5 × 10^−11^ m for rhodamine B (RhB), with an EF of 2.96 × 10^7^. This excellent SERS performance was attributed to the molecular enrichment provided by abundant active SERS regions in Gr MFs, along with synergistic effects from enhanced charge transfer and multiple light-scattering events. Similarly, in another study, MoS_2_@Ag nanoflowers (NFs)/rGO^[229]^ hybrid nanostructures were fabricated, exhibiting excellent SERS performance and multiplexed capability. Benefiting from the synergistic effects of numerous “hot spots” introduced by MoS_2_@Ag NFs, as well as the large molecular enrichment and CM effect of rGO, this SERS substrate achieved ultralow LODs of 4.7 × 10^−9^, 5.6 × 10^−9^, and 7.7 × 10^−8^ m for detecting melamine, VA, and BPA in milk, respectively. Notably, this sensing platform also possesses photocatalytic capability, enabling recyclable detection through in situ degradation of these mixed additives in milk. Ghopry et al.^[222]^ developed a novel vdWH composed of TMDC (MoS_2_ and WS_2_) nanodomes (NDs) and monolayer graphene, exhibiting excellent SERS performance comparable to that of metal NPs/graphene hybrids. Both CM and EM effects were enhanced, resulting from the increased electric dipole moment and dipole-dipole interactions at the vdW interface, as well as the LSPR near the TMDC NDs. Subsequently, the same group developed an Au NPs/WS_2_ NDs/graphene heterostructure^[191]^ for SERS-based biosensing (Figure 5e), achieving an order of magnitude higher sensitivity than the previous TMDC NDs/graphene system, owing to the superimposed LSPR effects from both WS_2_ NDs and Au NPs. This SERS substrate can be fabricated via layer-by-layer growth, indicating its potential for scalable and low-cost biosensor production. Later on, they fabricated intermixed WS_2_ and MoS_2_ nanodisks on graphene for sensitive SERS detection.^[230]^ Owing to the superposition of the LSPR effects, this sensing platform outperformed the SERS substrate based on a single type of TMDC on graphene. Zhao et al.^[164]^ fabricated a hybrid SERS substrate by integrating core-shell Au@Cu_2_O nanotriangles with Ti_3_C_2_T_x_ MXene ultrathin nanosheets. The system exhibited excellent SERS performance arising from the synergistic EM enhancement at the Au tips and across the entire nanotriangles, along with multiple charge-transfer pathways within the hybrid structures. Bian et al.^[231]^ fabricated a MOF@Ag/MXene hybrid structure on PDMS for the sensitive SERS detection of *E. coli* (Figure 6a). The overall high performance was attributed to the combined effects of CM enhancement, analyte enrichment, and EM amplification contributed by each component. In another work, Liu et al.^[232]^ developed a sensitive and flexible SERS sensor based on ZnO QDs/Ti_3_C_2_ MXene hybrids, as illustrated in Figure 6b. It achieved a LOD of 1 × 10^−7^ m for the detection of 4-mercaptopyridine (4-MPY) due to the combination of several effects: multiple charge transfer paths, improved charge-transfer efficiency facilitated by oxygen vacancies in ZnO QDs, and the presence of a Schottky barrier between Ti_3_C_2_ and ZnO QDs. Additionally, this biosensor demonstrated self-cleaning capability via photocatalysis and exceptional reusability in methylene blue (MB) detection, suggesting its strong potential for in situ molecular detection in food safety and environmental monitoring. Impressively, Li et al.^[223]^ fabricated Fe_3_O_4_@Au NPs/GO hybrid structures for rapid SERS detection and in situ monitoring of harmful polycyclic aromatic hydrocarbons (PAHs) in water, achieving a LOD of 3.8 μg L^−1^ for Benzo[a]pyrene (BaP) and μg L^−1^-level detection for other PAHs. The combination of excellent magnetic enrichment facilitated by Fe_3_O_4_, the strong adsorption capability of GO, and the pronounced LSPR effect of Au NPs enabled both sensitive SERS detection and photocatalytic removal of BaP, making it a promising approach for environmental monitoring and remediation.

Specially engineered nanostructures have also been actively explored for integration with 2DMs to expand their sensing capabilities. For example, Liang et al.^[224]^ fabricated Au nanopyramids/graphene hybrids as a label-free SERS platform. Combining it with principal component analysis (PCA) enabled precise differentiation and profiling of cell lines and cellular states. In another work, graphene-coated Au nanopyramids were designed, theoretically studied, and experimentally applied for label-free SERS sensing of AD biomarkers, offering rapid, quantitative, and high-sensitivity detection.^[225]^ More sophisticated nanostructures—such as Au NPs/graphene/Cu cone cavities^[226]^ and flexible graphene/Au NPs/rectangular pyramid PMMA hybrids^[227]^—were also fabricated as innovative SERS substrates for sensitive analyte detection. Extending beyond graphene, Miao et al.^[228]^ developed Ti_3_C_2_ MXene-coated Au/Ag bimetallic nanocuboids as SERS substrates for the multiplexed detection of CV, MG, and MB in natural pond water, achieving high sensitivity and accuracy. Table 4 provides a summary of recently reported 0D-2D hybrids with complex or specially engineered architectures for SERS sensing.

**Figure 7.**
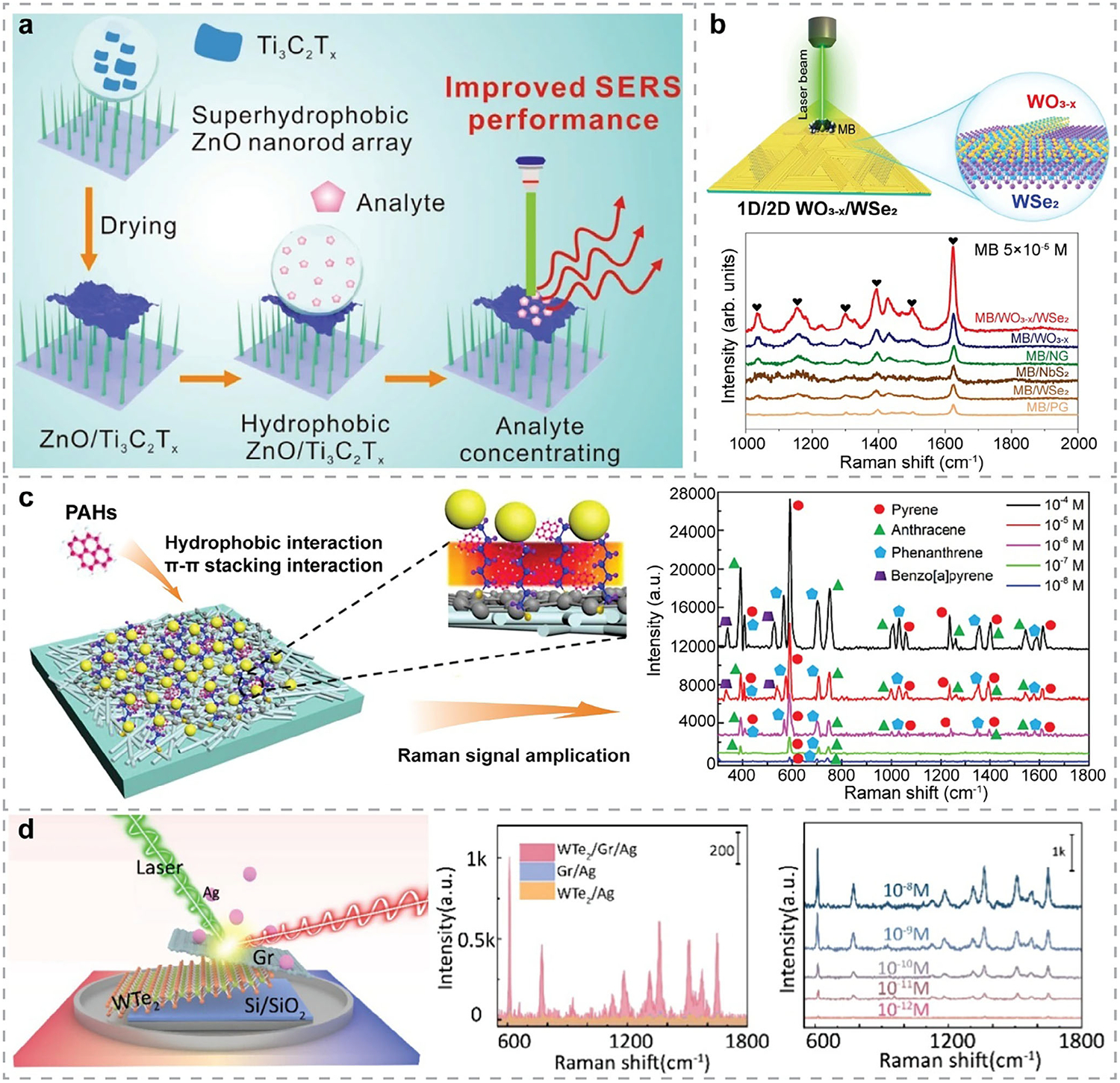
Recent advances in using 1D-2D and multicomponent hybrid nanostructures for SERS biosensing. a) Deposition of hydrophilic Ti_3_C_2_T_x_ on ZnO NRs for improved SERS performance. Reproduced with permission.^[236]^ Copyright 2023, Springer Nature. b) Fabricated 1D-2D WO_3-x_ NW/WSe_2_ heterostructures used as SERS substrate for attomolar-level molecular sensing. Reproduced with permission.^[237]^ Copyright 2023, Springer Nature. c) Developed metal–dielectric–metal hybrid nanostructures composed of Ag NPs/GO/Ag NWs as SERS sensors for PAH detection. Reproduced with permission.^[238]^ Copyright 2023, Elsevier. (d) Designed Ag NPs/graphene/WTe_2_ hybrid nanostructures with enhanced CM effect for SERS applications. Reproduced with permission.^[239]^ Copyright 2024, American Chemical Society.

1D nanostructures, such as NRs^[233–236]^ and NWs,^[237]^ have also been utilized in hybrid structures for SERS applications. For example, hBN/Ag NRs^[234]^ hybrid nanostructures were fabricated as recyclable substrates for label-free SERS detection of BR. Without sample pretreatment, this sensor achieved a LOD of 2.5 × 10^−8^ m for detecting BR in blood, owing to the strong affinity and high stability of hBN combined with the EM enhancement provided by Ag NRs. In another work, ultrathin Ti_3_C_2_ MXene nanosheets were decorated onto Ag NRs^[235]^ for sensitive on-site SERS detection of 3,3′,4,4′-tetrachlorobiphenyl (PCB-77) and 4-chlorobiphenyl (PCB-3), reaching LODs of 2.43 × 10^−10^ and 2.14 × 10^−9^ m, respectively. The effective protection of Ag NRs from oxidation by the MXene overlayer, along with the synergistic EM and CM enhancements within the MXene/Ag NRs hybrids, enabled multiplexed detection of PCBs in real soil samples with high sensitivity, stability, and reproducibility. MXene/ZnO NRs^[236]^ hybrid nanostructures were developed with potential for environmental pollutant monitoring (Figure 7a), in which the enhanced analyte affinity and strong charge transfer at the interface facilitated ultrasensitive SERS detection of R6G, achieving a LOD of 10^−11^ m. Additionally, this semiconductor nanoarray/2D MXene integration also demonstrated its potential for miRNA detection. More recently, 1D vdW NWs have also gained increasing interest in fabricating noble-metal-free SERS substrates with high sensitivity. For instance, Lv et al. synthesized 1D-2D WO_3-x_ NW/WSe_2_ heterostructures,^[237]^ as demonstrated in Figure 7b, which exhibited attomolar-level sensitivity for MB detection, with a LOD of 5 × 10^−18^ m. The exceptionally high EF of 5.0 × 10^11^ was attributed to ultrafast charge transfer induced by the NWs and strong interlayer coupling in the hybrid system. Table 5 summarizes recently reported 1D-2D hybrid nanostructures for SERS applications.

In 2014, Ling et al.^[75]^ investigated the Raman enhancement effects on different 2DMs. Since then, researchers have been exploring new 2DMs or stacking 2DMs together to create vdWHs for fabricating noble-metal-free SERS substrates with enhanced performance.^[240]^ Particularly, graphene/MoS_2_ vdWHs are among the most widely studied^[241]^ and have demonstrated significant potential in SERS-based biosensing applications. More recently, Wei et al.^[242]^ used first-principles DFT calculations to systematically investigate the wavelength-dependent SERS response of different 2DMs and of vdWHs with varying numbers of layers, where MoS_2_ and graphene exhibited strong and broadband enhancement effects. Experimentally, graphene/MoS_2_ vdWHs have been fabricated and utilized as label-free optical biosensors for detecting DOX,^[63]^ demonstrating good stability, sensitivity, and selectivity. More importantly, multidimensional imaging revealed the mechanisms controlling the biosensing performance, i.e., the doping level of MoS_2_ and graphene, the charge transfer between them, and the sources of non-uniform doping.^[63]^ More attractively, the SERS performance can be tuned by manipulating the doping state of exfoliated MoS_2_ monolayers, with n-doped MoS_2_/dopant hybrids demonstrating enhanced SERS signals for R6G detection.^[243]^ On the other hand, epitaxial graphene with the Fermi level modulated by intercalated 2D metals, i.e., atomically thin, air-stable metal layers fabricated by confinement heteroepitaxy (CHet),^[244]^ also exhibited improved GERS detection with higher sensitivity and selectivity.^[245]^ Meanwhile, the interlayer distance between MoS_2_ and graphene also influences the SERS performance,^[246]^ i.e., shorter distance enhances the dipole-dipole interactions and charge transfer, thereby increasing SERS efficiency. These insights offer valuable guidance for engineering 2D vdWHs through rational design of stacking configurations—such as stacking order and twist angles^[247]^—and tuning doping levels to maximize SERS performance. Although varying the number of layers in the heterostructure could optimize the SERS performance,^[240]^ calculations have shown that the topmost contact layer exerts the most significant influence within multilayer vdWH systems.^[242]^

Rationally designed multicomponent hybrid nanostructures with mixed dimensions have emerged to achieve synergistic enhancements in SERS performance. Since the undesired fluorescence background in SERS, originating from either analytes or substrates, can interfere with Raman signals, integrating materials that effectively quench fluorescence can significantly improve SERS performance. For example, in the design of hBN/graphene/Ag NPs hybrid structures for SERS detection,^[248]^ Cai et al. used graphene to suppress the fluorescence background from probe molecules. The SERS substrates can be regenerated by heating, while the hBN overlayer protects both graphene and Ag NPs from oxidation during this process. The multicomponent structures can also facilitate collective contributions from different mechanisms, further enhancing SERS performance. For instance, Lu et al.^[174]^ developed GO/Ag/3D PS nanospheres as SERS substrates for sensitive and multiplexed detection of melamine and dicyandiamide in dairy products. Jiang et al.^[249]^ fabricated Ag nanostars-multiwall carbon nanotube (MWCNT)/GO hybrids for nanoplastic detection. Owing to the EM enhancement of Ag nanostars, along with the CM and enrichment effects of MWCNT/GO, this SERS platform achieved a LOD of 5 × 10^−5^ mg mL^−1^ for detecting 50 nm PS nanoplastics. Wang et al.^[238]^ constructed metal-dielectric-metal nanostructures composed of Au NPs/GO/Ag NWs as SERS biosensors for identifying and detecting PAHs, as illustrated in Figure 7c. By leveraging the localized enhanced EM field generated between Au NPs and Ag NWs, along with the effective trapping of PAHs by GO through *π*–*π* interactions, this platform achieved LODs of 10^−8^ M for pyrene, anthracene, and phenanthrene, and 10^−7^ M for BaP, respectively, demonstrating excellent selectivity, sensitivity, and multiplexing capability. Similarly, Mei et al.^[173]^ developed Au/graphene/Ag/ZnO hybrid nanostructures that leveraged multidimensional plasmonic coupling for enhanced SERS performance. The graphene middle layer not only facilitated excellent analyte enrichment but also served as a protective barrier against Ag oxidation, which improved the overall stability and reproducibility of the system. In another work, Zhang et al.^[250]^ also exploited multidimensional plasmonic coupling of precious metals and fabricated a flexible Ag NPs/graphene/Cu hybrid SERS substrate with high performance. Notably, Sun et al.^[239]^ designed Ag NPs/graphene/WTe_2_ hybrid nanostructures on a Si/SiO_2_ substrate (Figure 7d). By leveraging the energy-level alignment between WTe_2_ and graphene, the charge transfer process was promoted, resulting in a significantly enhanced CM effect in SERS. This system exhibited substantially higher SERS performance than Ag NPs/WTe_2_ or Ag NPs/graphene, with the LOD reduced by three orders of magnitude for R6G, providing insights into the rational design of highly sensitive SERS platforms with stronger CM contribution. MXenes have also been integrated into multicomponent nanostructures. For instance, Liu et al.^[251]^ developed Au NPs/MoS_2_/MXene hybrids for miRNA detection, achieving a linear range of 10 am–1 nm and a LOD of 6.61 am, indicating the strong potential of multicomponent nanohybrids for ultra-sensitive SERS sensing. Table 6 summarizes recently reported multicomponent nanohybrids with mixed dimensions for SERS applications.

### 5.2. Recent Advances in SPR Biosensing

**Figure 8.**
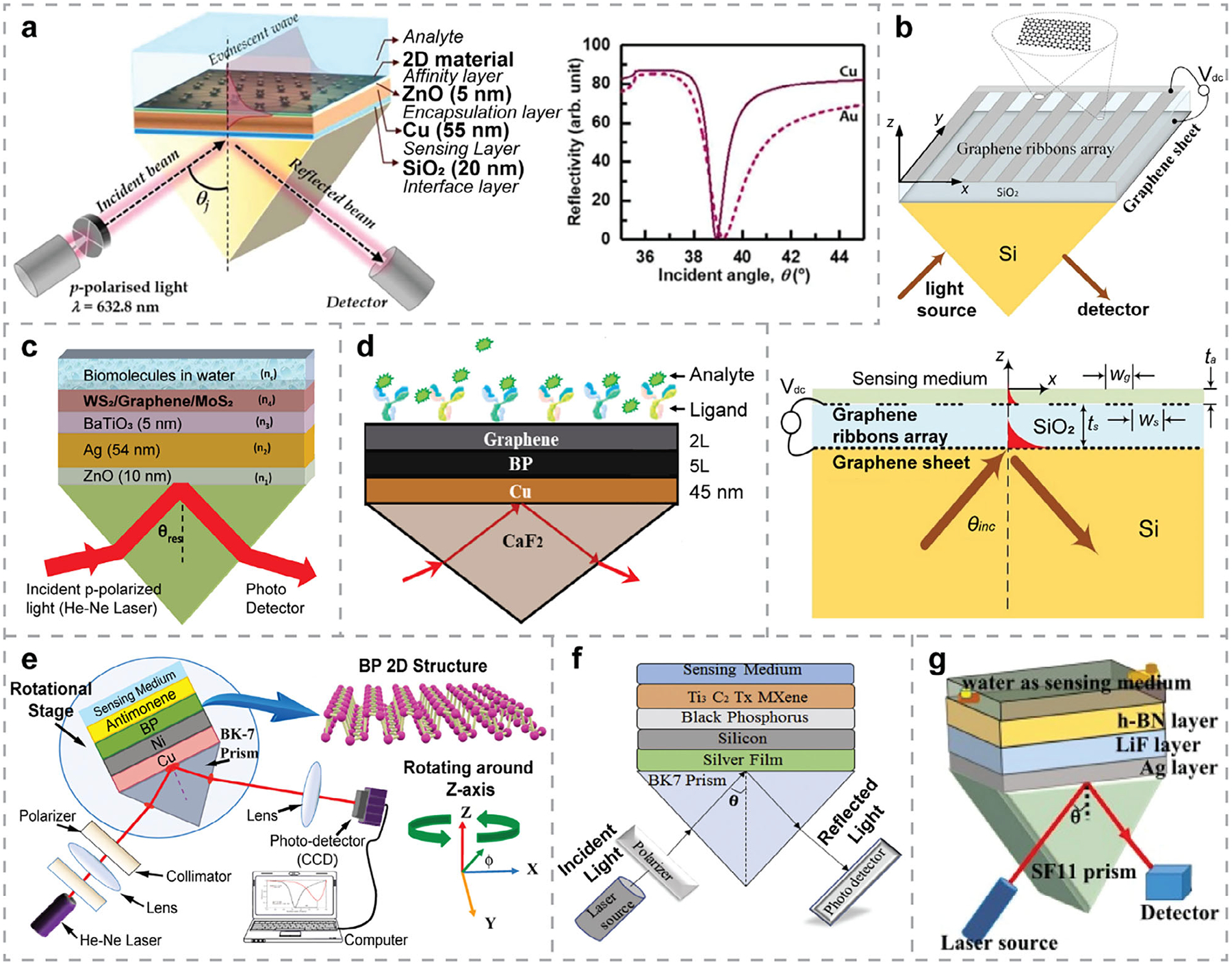
Recent advances in using hybrid nanostructures for SPR biosensing with Kretschmann configuration. a) Strategies to enhance performance and commercial feasibility of copper-based SPR sensors through multilayer structural design. Reproduced with permission.^[255]^ Copyright 2024, Elsevier. b) Proposed evanescent-wave SPR biosensor based on gate-controlled graphene nanoribbons array. Reproduced with permission.^[256]^ Copyright 2021, Springer Nature. c) Comparative performance study of multilayer SPR biosensors integrating different 2DMs (WS_2_, MoS_2_, and graphene). Reproduced with permission.^[257]^ Copyright 2020, Elsevier. d) Graphene/BP-integrated SPR biosensor proposed for in situ monitoring of SARS-CoV-2 Omicron. Reproduced with permission.^[258]^ Copyright 2023, Elsevier. e) Integration of antimonene/BP heterostructure with Ni/Cu-based SPR biosensor for enhanced sensitivity. Reproduced with permission.^[259]^ Copyright 2021, Elsevier. f) Proposed SPR biosensor composed of Ti_3_C_2_T_x_ MXene/BP/Si/Ag/BK7 prism with improved sensitivity. Reproduced with permission.^[260]^ Copyright 2020, Elsevier. g) Ultrasensitive Fano-resonance SPR sensor based on hBN/LiF/Ag/SF11 multilayer structure. Reproduced with permission.^[261]^ Copyright 2025, Elsevier.

SPR biosensors offer significant advantages for real-time monitoring of molecular interactions and analyte binding without the need for labeling. Since the working principle of SPR biosensors relies on the excitation of surface plasmons, metallic thin films (such as Au and Ag), which possess prominent plasmonic properties in the visible regime, are crucial components in the sensor design. Au thin films are particularly favorable due to their chemical inertness and robustness in supporting surface plasmons. However, Au does not adsorb bioanalytes efficiently, limiting the sensitivity of conventional SPR biosensors. Ag thin films, while exhibiting stronger plasmonic behavior than Au and enhancing the sensitivity, i.e., with SPR peaks of greater intensity and narrower width, are prone to oxidation and corrosion,^[252]^ which affects the stability of SPR biosensors. To overcome these limitations, other metals have been explored, with Cu emerging as a compelling alternative due to its similar plasmonic behavior to Au but with higher electrical conductivity.^[253,254]^ The Cu-based SPR biosensor exhibited a narrower SPR peak width and a lower minimum reflectance value compared to that of the Au-based sensor (Figure 8a).^[255]^ Additionally, Cu is more cost-effective and feasible for scalable fabrication. Beyond finding substitute metals, researchers have also explored multilayer designs to improve sensor performance. As illustrated in Figure 8a, each layer serves its specific function in the Kretschmann configuration, such as an affinity layer for binding events, an encapsulation layer above the sensing layer for protection, and an interface layer between the prism and the sensing film for better adhesion. The choice of different materials for these layers significantly impacts the overall sensor performance, which has been systematically evaluated.^[255]^

When integrating 2DMs with SPR biosensors, the plasmonic properties and thereby the sensor performance can be further tuned. The large surface area of 2DMs supports numerous active sites, ensuring efficient bioanalyte adsorption. Covering metallic thin films with 2DMs can also prevent oxidation and corrosion, thus improving the durability of SPR biosensors. Additionally, the surface of 2DMs can be functionalized with different molecules or complementary target analytes, providing selective and sensitive detection for various applications. In particular, 2DMs facilitate strong light–matter interactions, which could promote the coupling between incident light and surface plasmons. Moreover, the tunability of the electrical and optical properties of 2DMs allows further tailoring of the SPR properties to suit specific purposes. By stacking 2DMs together into multilayer systems, more possibilities arise for enhancing the light-matter interaction, particularly by increasing the optical absorption of the system and thereby improving the performance of SPR biosensors. In this subsection, we review recent advances in SPR biosensors that benefit from 2DMs and their hybrid structures, discussing the roles of individual 2DMs as well as their synergistic effects in enhancing sensor performance.

#### 5.2.1. SPR Biosensors Based on Kretschmann Configuration

Graphene has been widely used to enhance the performance of SPR biosensors. Due to *π*-*π* interactions, its strong surface affinity enables efficient binding of bioanalytes, thereby improving the detection capability. In addition, its high carrier mobility and strong optical absorption can enhance the responsiveness of SPR biosensors to the surface binding events, consequently increasing sensitivity and enabling real-time monitoring. Moreover, the optical properties of graphene can be tuned by electrical gating,^[262]^ strain engineering, or doping, offering greater flexibility in optimizing sensor performance. For instance, Chung et al.^[263]^ reported an SPR biosensor with improved sensing performance by depositing graphene on an Ag thin film and uncovered the mechanism underlying this enhancement. By measuring the work function of graphene/Au with varying numbers of graphene layers and doping levels, they deduced that the sensitivity enhancement was attributed to the surface dipole induced by the charge transfer between graphene and Au. Notably, they observed less sensitivity enhancement with three layers of graphene than with monolayer or bilayer graphene, which was explained by the damping of surface plasmons. This phenomenon was also observed in another study, where Panda et al.^[264]^ investigated the performance of a graphene/Au-based SPR biosensor for glucose and gas detection. A nearly twofold increase in the electric field intensity was observed in the optimized structure with monolayer graphene on 55 nm Au, compared to a conventional Au-based SPR biosensor without graphene. However, as the number of graphene layers increased, the SPR curves became broader and shallower, due to the damping of surface plasmons by graphene. Given that more graphene layers could potentially improve the optical absorption, these collective findings suggest that one should carefully consider the competing effects and balance the number of graphene layers to achieve optimal sensor performance. In other works, graphene was also reported to serve as a protective layer for Ag thin films,^[69,265]^ improving both the sensitivity and stability of SPR biosensors. More recently, Jena et al.^[266]^ proposed an SPR biosensor with graphene/AlN/Ag/TiO_2_/BK7 prism structure. By optimizing the thickness of each constituent layer through simulation, they achieved sensitivities of 138.46 deg RIU^−1^, 163.63 deg RIU^−1^, and 182.85 deg RIU^−1^ for detecting infected plasma, platelets, and hemoglobin, respectively, indicating the potential of SPR biosensors for rapid and high-sensitivity detection of pathogens. In another work, graphene was used in a proposed SPR biosensor for detecting the SARS-CoV-2 virus.^[267]^ Upon optimization, a maximum sensitivity of 433.63 deg RIU^−1^ was achieved using monolayer BaTiO_3_ and bilayer graphene within the thiol-tethered ssDNA/graphene/BaTiO_3_/Ag/TiO_2_/CaF_2_ prism structure.

Beyond 2D graphene, fabricating a periodic array of 1D graphene nanoribbons can more effectively couple incident light to surface plasmons. Hwang^[256]^ computationally designed an SPR biosensor based on gate-controlled graphene nanoribbons, as illustrated in Figure 8b. Notably, owing to the localized enhanced field at the nanoribbon edges, the binding of target analytes significantly modulates the SPR frequency, thereby enabling sensitive and metal-free SPR detection. Unlike conventional SPR configurations, this device generates the SPR curve by sweeping the graphene chemical potential while maintaining a fixed incident angle. For analytes with a RI of 1.33, the calculated sensitivity reached 36 401.1 mV RIU^−1^. In another work, Barrios^[268]^ investigated the optical response and sensitivity of a graphene nanoribbon/Au-based SPR biosensor integrated with a microfluidic flow cell. Nanoribbons with a high height-to-width aspect ratio were found to be optimal for minimizing the LOD.

As an analog to graphene but with superior physical and chemical properties, antimonene has recently emerged and attracted great interest in enhancing the performance of SPR biosensors.^[71]^ In addition to high hydrophilicity and large surface area, the high adsorption energy and large work function make antimonene preferable as an affinity layer compared to other 2DMs. For example, an Au NRs/antimonene-based SPR biosensor^[88]^ achieved label-free, molecular-level quantification of miRNA with a LOD of 10 am. In another study, Singh et al.^[142]^ proposed an SPR biosensor with antimonene/Au/BK7 prism structure for miRNA detection. It exhibited a sensitivity of 181.9 deg RIU^−1^, outperforming both graphene/Au-based (147.95 deg RIU^−1^) and conventional Au-based SPR biosensors (144.45 deg RIU^−1^). Notably, three layers of antimonene led to a less pronounced improvement in sensitivity, similar to the observed phenomenon with graphene layers, which was attributed to the electron energy loss. However, since the imaginary part of its dielectric constant is small, antimonene exhibits much less energy loss compared to graphene. These antimonene-based SPR biosensors are not only promising for real-time detection of hybridization events, but also demonstrate strong potential for practical applications in early cancer diagnosis. Beyond miRNA detection, antimonene-based SPR biosensors have also been proposed for detecting other analytes^[146,147]^ with high sensitivity. For instance, Singh et al.^[147]^ designed an antimonene/BaTiO_3_/Ag/BaF_2_ prism SPR biosensor for hemoglobin detection, achieving a sensitivity of 303.83 deg RIU^−1^, with antimonene serving as a highly efficient biomolecular recognition layer.

Besides graphene, TMDCs have also been utilized to enhance SPR biosensors due to their higher optical absorption. Kumar et al.^[257]^ conducted a comparative study of WS_2_-, MoS_2_-, and graphene-covered BaTiO_3_/Ag/ZnO/BK7 prism SPR sensors (Figure 8c). Among them, WS_2_ exhibited the highest sensitivity (180 deg RIU^−1^), outperforming MoS_2_ (174 deg RIU^−1^) and graphene (157 deg RIU^−1^). Although the graphene/BaTiO_3_/Ag/ZnO/BK7 prism biosensor showed an SPR peak with a narrower full width at half maximum (FWHM), along with higher quality factor and detection accuracy, the highest optimized sensitivity (235 deg RIU^−1^) was achieved with the WS_2_ (bilayer)/BaTiO_3_/Ag/ZnO/BK7 prism configuration. In another work, Ouyang et al.^[269]^ studied the enhancement effect of various TMDCs (MoS_2_, MoSe_2_, WS_2_, and WSe_2_) integrated with a Si thin film in conventional Au-based SPR biosensors, where these additional dielectric layers served as effective light-absorbing media. The topmost TMDC layer was in direct contact with target bioanalytes, acting as an affinity layer. Additionally, the induced charge transfer from the TMDC to Au enhanced the overall SPR performance. The highest sensitivity was achieved using the WS_2_ (monolayer)/Si (7 nm)/Au (35 nm) configuration. More recently, Daher et al.^[270]^ theoretically investigated an Ag-based SPR biosensor coated with WSe_2_/Si for detecting urea in blood, achieving a maximum sensitivity of 373.49 deg RIU^−1^ after optimization. Various TMDC layers, such as WS_2_^[271]^ and WSe_2,_^[272]^ have also been employed as highly effective biomolecular recognition layers, further enhancing SPR sensor performance. Dey et al.^[273]^ designed a WSe_2_/Al/Ag SPR biosensor with improved performance. They attributed the enhanced sensitivity to the increased optical absorption arising from the large number of excited electrons at the metal/WSe_2_ interface, as indicated by the electric field intensity peak at the WSe_2_/analyte interface.

MXenes also play an important role in enhancing SPR performance. They support surface plasmon polaritons and can amplify the plasmonic resonance when integrated with metal thin films. Notably, the plasmonic properties of MXenes can be tuned by modifying their surface functional groups.^[80]^ Besides, the charge transfer between MXenes and metal thin films can further modulate the electronic and optical properties at the interface, contributing to the development of highly sensitive and tunable SPR biosensors. Additionally, the MXene layer coated on top of Ag film can act as a protective barrier against oxidation.^[274–276]^ Moreover, MXenes can serve as effective biomolecular recognition layers in SPR biosensors, enhancing the sensing capabilities.^[79,277]^ Gan et al.^[128]^ developed a 2D Ti_2_C MXene-based SPR biosensor, the performance of which strongly depended on the thickness of Ti_2_C nanosheets. The optimized design showed a 79% improvement in sensitivity compared to an SPR sensor without the Ti_2_C overlayer. This biosensor also demonstrated its ability to detect heavy metal ions, achieving LODs of 79.2, 56.5, and 92.8 ng L^−1^ for Pb^2+^, Cr^2+^, and Hg^2+^ ions, respectively.

BP, with good hydrophilicity and a large surface-to-volume ratio, can not only act as an affinity layer for bioanalytes, but its layer-dependent bandgap also enables strong and tunable optical absorption, thus enhancing the electric field intensity at the interface when integrated with metal thin films.^[278–281]^ For example, in a BP/Ag-based SPR biosensor,^[278]^ the presence of the BP layer facilitated strong light-matter interaction, leading to improved sensor performance. Bouandas et al.^[279]^ proposed an SPR biosensor with BP/Ni/SiO_2_/Cu/BK7 prism structure, in which the superior properties of BP significantly enhanced the sensitivity. In another study, Karki et al.^[281]^ compared the performance of SPR biosensors incorporating different metal thin films (Au, Ag, Cu) in the BP/metal/BaTiO_3_/metal/BK7 prism structure, and observed a significantly increased electric field intensity at both the BP/Cu and sensing medium/BP interfaces. However, despite these promising results, the major drawback limiting the practical application of BP in SPR biosensors is its instability in both air and water.

Covering BP with other 2DMs can prevent its oxidation, which improves the overall stability of SPR biosensors. Additionally, such hybrid structures may further enhance sensitivity.^[143,144,259,282]^ For instance, Akib et al.^[283]^ systematically compared different 2DM combinations within an Ag-based SPR biosensor designed for SARS-CoV-2 detection. Among these, graphene/BP exhibited the highest sensitivity of 390 deg RIU^−1^, along with the best detection accuracy. Similarly, in a later study, they presented the most effective configuration integrating graphene/BP with a Cu-based SPR biosensor for real-time monitoring of the SARS-CoV-2 Omicron variant (Figure 8d),^[258]^ where an enhanced sensitivity of 410 deg RIU^−1^ was obtained. The achieved performance improvements in these graphene/BP-integrated SPR biosensors are attributed to: i) the high optical absorption of BP; ii) the enhanced electric field resulting from charge transfer between the graphene/BP heterostructure and the metal thin film; and iii) the strong adsorption of bioanalytes on graphene via *π*–*π* interactions. In another study, Yuan et al.^[282]^ designed an Au-based SPR biosensor with a few layers of BP covered by monolayer graphene. While increasing the number of BP layers improves optical absorption, excess layers can lead to optical loss and plasmon damping. Therefore, the optimal sensing performance was achieved by carefully balancing the Au film thickness and the number of BP layers. Notably, considering that anisotropic BP acts as an optical polarizer, the sensitivity was further improved through optimization of the rotation angle of the proposed SPR sensor.

Besides graphene, the outstanding stability of antimonene also makes it a desirable protective layer in SPR biosensors.^[143,144,259]^ For example, an antimonene/BP-based SPR biosensor was proposed for detecting DNA hybridization.^[143]^ This design not only facilitated strong charge transfer between antimonene/BP and the Al thin film, thereby enhancing SPR sensitivity, but the antimonene overlayer also effectively protected BP from degradation, thus improving the stability of the proposed biosensor. In another study, Singh et al.^[259]^ presented a high-performance Ni/Cu-based SPR biosensor that integrated an antimonene/BP heterostructure, as illustrated in Figure 8e. In addition to the strong charge transfer between antimonene/BP and the bimetallic Ni/Cu thin films, as the number of BP layers increased, higher carrier mobility was obtained due to the reduced effective mass, resulting in significantly enhanced sensitivity. Moreover, by leveraging the anisotropic optical properties of BP, the sensitivity of the designed sensor was further optimized through rotation-angle tuning. Consequently, an ultrahigh sensitivity of 446.90 deg RIU^−1^ was achieved using five BP layers covered by monolayer antimonene, outperforming all other 2DM-based SPR biosensors at that time. The monolayer antimonene not only allowed effective binding of bioanalytes but also acted as a passivation layer protecting BP against oxidation.

Graphene/TMDC is another widely used heterostructure in SPR biosensors. The use of graphene alone for signal enhancement is limited due to its relatively low optical absorption compared to TMDCs,^[284,285]^ while the inherent instability of TMDCs also constrains the performance of SPR biosensors. To address these issues, researchers have explored graphene/TMDC heterostructures to enhance overall sensor performance^[284]^ and minimize device size.^[285]^ For instance, Rouf et al.^[286,287]^ proposed a graphene/MoSe_2_/Ag/Ti/BK7 prism SPR biosensor for detecting various bioanalytes, including DNA hybridization events. A maximum sensitivity of 215.5 deg RIU^−1^ was reported, which is 2.42 times higher than that of graphene/Au-based SPR biosensors. In another work, Kumar et al.^[288]^ conducted a comparative study of the SPR performance based on three different designs, i.e., graphene, graphene/MXene, and graphene/MoS_2_, on Ag/Ti/BK7 prism structure, for the detection of carcinoembryonic antigen (CEA) biomarkers. They found that the integration of graphene/MoS_2_ provided the greatest enhancement in overall sensor performance.

Heterostructures composed of MXenes and other 2DMs have also been reported to enhance SPR performance. For example, the designed Ti_3_C_2_T_x_/graphene-based SPR sensor^[289]^ demonstrated its highest sensitivity of 241.20 deg RIU^−1^ when using three layers of MXene. In another study, an antimonene/Ti_3_C_2_T_x_ hybrid structure^[290]^ was employed in an Au-based SPR biosensor, where a sensitivity of 224.26 deg RIU^−1^ was achieved, owing to the high conductivity of MXene and the superior properties of antimonene. Kumar et al.^[291]^ proposed an SPR biosensor using a Ti_3_C_2_T_x_/BP hybrid structure on bimetallic Ni/Cu thin films, achieving a maximum sensitivity of 304.47 deg RIU^−1^. In another work, they designed a similar SPR biosensor^[260]^ using an Ag thin film instead, with an additional Si layer inserted between the Ti_3_C_2_T_x_/BP heterostructure and the Ag film (Figure 8f). This design exhibited a significantly improved sensitivity of 127.58% over that of a conventional Au-based SPR sensor, owing to: i) the high metallic conductivity, biocompatibility, hydrophilicity, and strong surface affinity of functionalized Ti_3_C_2_T_x_;^[260,291]^ ii) the high charge carrier mobility and carrier confinement,^[260]^ as well as the layer-dependent direct and tunable bandgap of BP;^[291]^ and iii) the integration of a high-RI Si layer.^[260]^ The heterostructure of MXene/BP could provide higher charge carrier mobility, and these layered materials also facilitate strong light-matter interactions, which consequently increase the overall sensitivity of SPR biosensors.

By constructing heterostructures of 2DMs, additional coupling effects can be introduced into the system, further enhancing sensitivity. For instance, Kumar et al.^[261]^ proposed a Fano resonance-based SPR biosensor with hBN/LiF/Ag/SF11 prism structure (Figure 8g), achieving a phase sensitivity of 1000 deg RIU^−1^ and a wavelength sensitivity of 900 nm RIU^−1^. The Fano resonance was produced by the coupling between surface plasmon polaritons and the photonic waveguide modes supported by 2D hBN.^[261]^ This Fano resonance introduces a sharp and steep feature, allowing for much more sensitive detection compared to standard SPR systems. In another work, Wang et al.^[292]^ designed a tunable Fano resonance-enhanced SPR biosensor based on MXene/MoS_2_ heterostructure. An optical waveguide layer was introduced to support planar waveguide modes. These modes coupled with the SPR, which was enhanced by the MXene/MoS_2_ heterostructure. This effective coupling substantially improved the responsiveness of the biosensor to bioanalytes and enhanced the overall sensitivity.^[292]^

Integrating multilayer heterostructures of 2DMs into SPR biosensors not only allows for further tuning of the system but also enables detection across a broader range of RIs, accommodating diverse biological samples.^[293,294]^ For instance, an SPR biosensor with blue phosphorus-MoS_2_/Au/PtSe_2_/CaF_2_ prism structure^[293]^ was capable of detecting across a broad RI range from 1.33 to 1.36; another sensor with WS_2_/HfSe_2_/BP/Ag/BAK1 prism^[294]^ configuration could detect across an extended RI range from 1.33 to 1.39. Ghodrati et al.^[295]^ proposed a multilayer SPR biosensor composed of Ti_3_C_2_T_x_/MoO_3_/MoS_2_/Ag/BK7 prism aimed at detecting glucose in aqueous solutions with varying RIs. Table 7 summarizes recent 2DM- and their hybrid nanostructure-based SPR biosensors with the Kretschmann configuration.

The use of multilayer 2DM structures also enables the miniaturization of SPR biosensors for practical applications. In particular, optical fiber-based SPR biosensors are favored due to their compact size and flexibility for in situ and remote sensing. Additionally, optical fibers can withstand complex and harsh environments. From an application perspective, fiber-optic SPR biosensors have demonstrated versatility in both environmental monitoring and medical diagnostics. In the next subsection, we provide an overview of recent advances in fiber-optic SPR biosensors utilizing 2DMs and their hybrid nanostructures.

#### 5.2.2. Optical Fiber-Based SPR Biosensors

**Figure 9.**
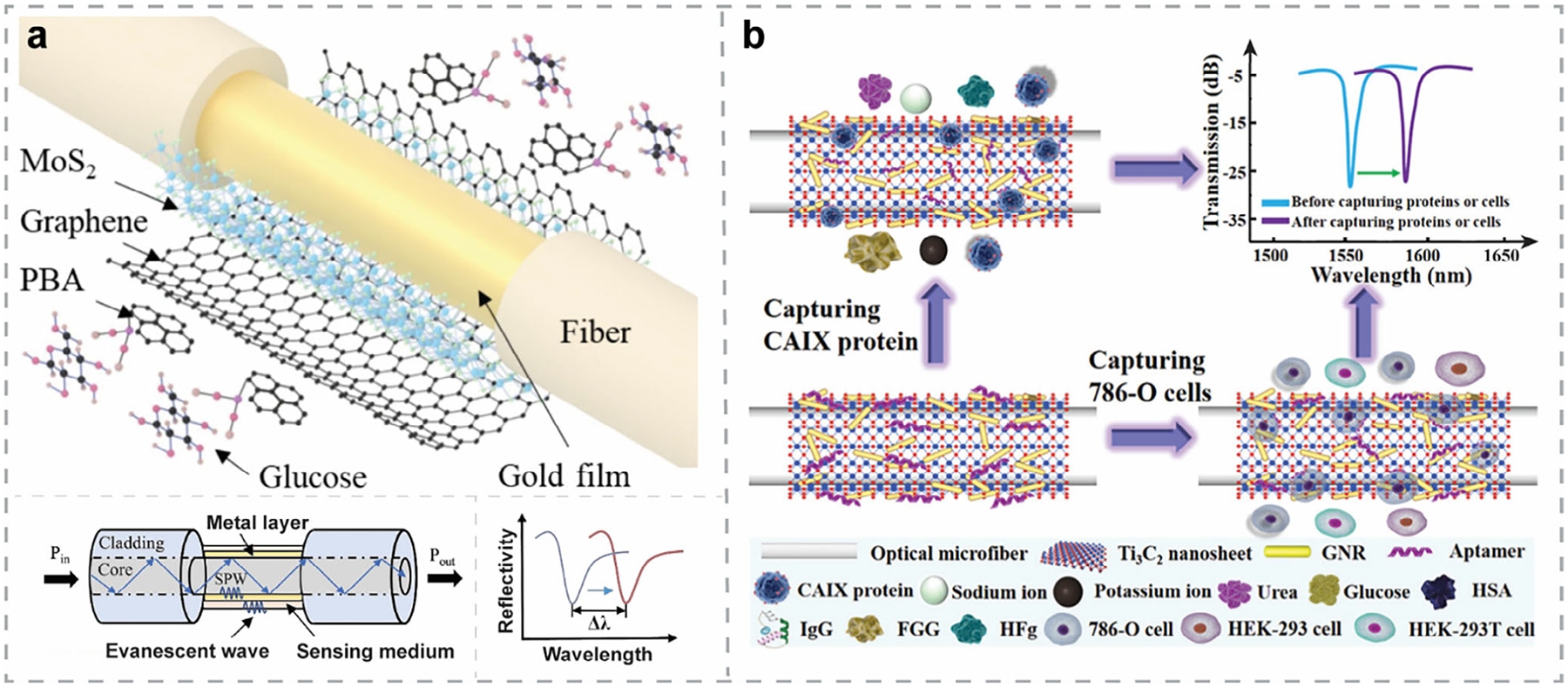
Recent advances in using hybrid nanostructures for optical fiber-based SPR biosensing. a) Designed and fabricated fiber-optic SPR biosensor utilizing graphene/MoS_2_ heterostructure for highly sensitive glucose detection. Reproduced with permission.^[298]^ Copyright 2025, Elsevier. b) Functionalized optical fiber SPR biosensor based on Ti_3_C_2_ MXene/Au NRs for ultrasensitive detection of renal cancer proteins and cells. Reproduced with permission.^[299]^ Copyright 2023, American Chemical Society.

Fiber-optic SPR biosensors are well known for their advantages in miniaturization, flexibility, and the ability to remotely monitor bioanalytes. However, due to the limited sensing area, their sensitivity is generally lower compared to prism-coupled SPR biosensors. To address this limitation, researchers have explored the integration of 2DM hybrids to enhance sensor performance. For example, Rahman et al.^[296]^ proposed a highly sensitive optical fiber-based SPR biosensor integrating graphene/MoS_2_ hybrid structure with Ag for the detection of DNA hybridization. Numerical modeling showed that the designed sensor achieved a high sensitivity of 105.71 deg RIU^−1^ and a detection accuracy of 1.626. In another work, Rahman et al.^[297]^ theoretically studied the performance of a fiber-optic SPR biosensor integrating 2DM/BP with Ag, where maximum improvement was achieved using 10 layers of BP covered with 3 layers of graphene. Recently, Vikas et al.^[145]^ designed a tapered fiber-optic SPR biosensor composed of antimonene/graphene/Au for the detection of cancerous cells. Owing to the effective charge transfer from antimonene/graphene to Au, and the strong optical absorption of antimonene/graphene, the sensitivity was significantly enhanced. Through numerical simulations, a sensitivity of 7.3465, 10.9250, 11.8914, and 15.2414 μm RIU^−1^ was obtained for sensing skin, cervical, blood, and adrenal gland cancer, respectively, indicating the potential application of this biosensor in early-stage cancer diagnosis. More recently, Ma et al.^[298]^ developed a fiber-optic SPR biosensor integrating graphene/MoS_2_ with Au/Cr thin films (Figure 9a) for the sensitive detection of glucose. This heterostructure-modified biosensor exhibited a 9.11-fold increase in sensitivity compared to the Au-based optical fiber SPR sensor, achieving a sensitivity of up to 12 593.06 nm RIU^−1^ for glucose concentrations ranging from 0–500 mg dL^−1^, along with excellent selectivity. These results highlight the strong potential of this optical fiber-based SPR sensor for continuous glucose monitoring.

Hybrid structures of 2DMs and NPs have also been demonstrated to enhance fiber-optic biosensors based on LSPR, and have been widely investigated by researchers. Li et al.^[300]^ reported a double S-tapered optical fiber-based LSPR biosensor using Au NPs coated with either Nb_2_CT_x_ MXene or GO for comparison. The detection sensitivities of tyramine were 17 and 34 pm μm^−1^ across a concentration range of 0–300 μm, respectively, with the superior performance of Nb_2_CT_x_ MXene/Au NPs attributed to the abundant functional groups on the Nb_2_CT_x_ surface. Subsequently, the same group developed a W-shaped fiber-optic LSPR biosensor based on Nb_2_CT_x_/Au NPs^[301]^ for the detection of tyramine, achieving a LOD of 6.96 μm. The sensor also exhibited high specificity, good stability, repeatability, and reusability. Impressively, Li et al.^[299]^ functionalized the optical fibers with Ti_3_C_2_ MXene/Au NRs for the ultrasensitive detection of renal cancer proteins and cells (Figure 9b). This biosensor achieved an ultralow LOD of 13.8 zm in pure buffer solution and 0.19 am in 30% serum solution. Additionally, it successfully identified live renal cancer cells in culture media with a LOD of 180 cells mL^−1^ and high specificity. These findings highlight the potential of this sensing platform for early-stage cancer biomarker detection. More recently, Zhang et al.^[302]^ demonstrated the clinical potential of a designed optical fiber-based LSPR biosensor for the rapid and accurate detection of neurotransmitters. Based on Ti_3_C_2_ MXene/Au NRs, it achieved a high sensitivity for acetylcholine with a LOD of 4.42 μm. Table 8 summarizes recent 2DM hybrid nanostructure-integrated fiber-optic SPR biosensors.

### 5.3. Toward Multifunctional Platforms: Multiplexing, Multimodal, and ML

The rational design of mixed-dimensional hybrid nanostructures based on 2DMs and novel nanomaterials not only provides an approach for improving the performance of biosensors, such as the synergistic enhancements for SERS, but also enables the development of biosensing platforms with complex functions, including multiplex and multimodal detection.

Simultaneous detection of multiple analytes within one single test is more efficient and offers higher throughput to provide a comprehensive understanding of the tested system, which is essential in medical diagnostics and environmental monitoring. However, multiplexing often demands higher performance of the biosensor in sensitivity, selectivity, and reliability. Thus, scientists have explored different strategies in developing multiplexed biosensing platforms, one of which is the employment of mixed-dimensional hybrid nanostructures based on 2DMs. For instance, a SERS substrate consisting of Au/Ag bimetallic nanocuboids coated with Ti_3_C_2_ nanosheets^[228]^ enabled the highly sensitive and reproducible identification of multiple fish drug residues in pond water. The GO-coated Ag-Au nanostars^[219]^ were used as a solution-based SERS biosensing platform for the ultrasensitive and multiplex detection of pesticides, exhibiting high stability and reproducibility. The MXene/Ag NRs substrate^[235]^ was developed to perform multiplex biosensing of PCBs in real soil samples with high sensitivity, reliability, and good recovery percentages, indicating its great potential in simultaneous SERS detection of multiple pollutants at the point of need. The developed GO/Ag/3D PS nanospheres hybrid SERS substrate^[174]^ has demonstrated multiplexed capability in the detection of melamine and dicyandiamide in dairy products. Ag NPs/GO/Ag NWs^[238]^ hybrids were explored as SERS-based biosensors for identifying and multiplexed detection of PAHs. These hybrid nanostructures are very powerful in sensitive and selective detection of target analytes in complex sample matrices, without compromising their reliability and stability. In another work, a robust SERS sensor was fabricated using MXene/GO fibers^[303]^ for the rapid, multiplexed, and label-free detection of pesticide residues. Besides SERS, SPR biosensors can also be developed for multiplex detection. When integrated with microfluidics, the SPR-based platforms not only offer real-time detection but also enable multiplexed biosensing with high throughput.^[304–306]^

**Figure 10.**
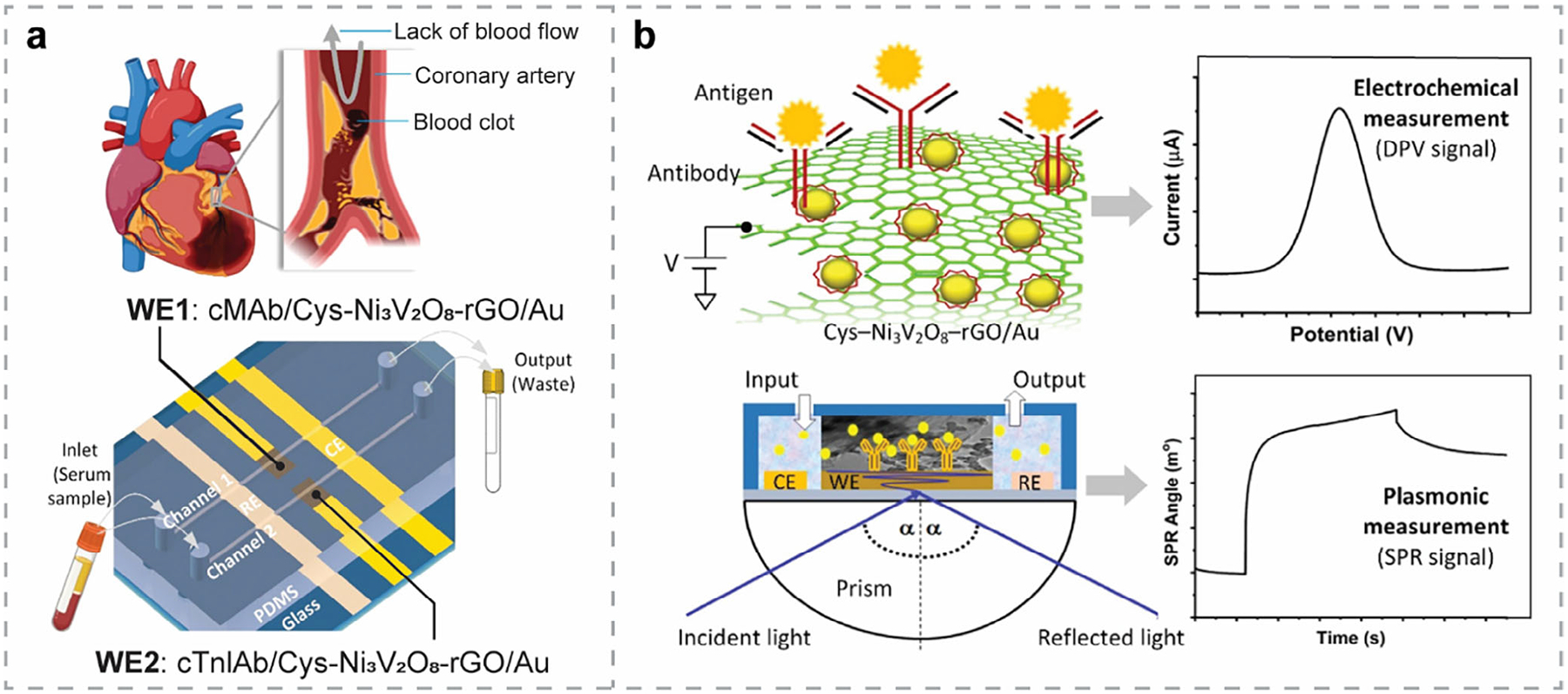
Electrochemical and SPR detection of myocardial infarction using microfluidic biochip based on hybrid nanostructures. a) Schematics of myocardial infarction (heart attack) (upper panel) and the designed microfluidic device (lower panel). b) Dual-modal detection capability of the developed biosensing platform. Reproduced with permission.^[309]^ Copyright 2024, American Chemical Society.

In addition to multiplexing, scientists have also developed biosensing platforms capable of multimodal detection. For instance, the graphene/MoS_2_ heterostructure was used for multimodal detection of DOX.^[63]^ The PL of MoS_2_ was modulated by the existence of DOX, and with the help of GERS, the Raman signal of DOX was significantly enhanced. This PL-GERS combination enabled more accurate and reliable detection. In another work, an SPR-SERS plasmonic sensor was developed based on graphene/Au NPs hybrid nanostructures,^[307]^ for the sensitive and dual-modal detection of pesticide residues in soil. This multifunctional sensing platform provided more options for detecting and verifying data, improving both the reliability and repeatability of the detection. Furthermore, optical biosensors can also be integrated with electrochemical detection to form a dual-modal sensing platform. For instance, Kim et al.^[308]^ fabricated a multifunctional biosensor based on GO/MoS_2_ hybrid nanocomposites capable of detecting Middle East respiratory syndrome coronavirus (MERS-CoV). This biosensor supports both electrochemical and SERS sensing, providing highly sensitive and selective detection of MERS-CoV. Very impressively, utilizing Ni_3_V_2_O_8_ nanospheres/rGO/Au hybrid nanostructures, Singh et al.^[309]^ developed a microfluidic device, as illustrated in Figure 10a, for the rapid and dual-modal (electrochemical and SPR) detection (Figure 10b) of cardiac myoglobin (cMb) and cardiac troponin I (cTnI), with pg mL^−1^-level LODs. This sensing platform enables real-time monitoring of the dynamic antibody-antigen interactions, and facilitates self-validation through dual modes, which reduces false readouts. The combination of electrochemical and SPR biosensing complements the advantages of both techniques, providing a multifunctional sensing platform with both high sensitivity and superior tracking of molecular interactions. This innovative sensing platform demonstrated its potential for cardiovascular disease management and monitoring other clinically important biomolecules. In another work, Zhu et al.^[203]^ developed a bifunctional nanosensing platform based on Ti_2_C MXene/Au-Ag nanoshuttles hybrid structures. The dual-modal detection, combining electrochemical response and SERS, facilitated accurate determination of carbendazim (CBZ) residues in tea and rice, with improved reliability through cross-validation of the two methods. Additionally, ML models were developed to perform intelligent analysis of both electrochemical and SERS data.

ML is very powerful in building algorithms that allow computers to learn patterns from a large dataset without being explicitly programmed for each specific task. Particularly, ML algorithms are useful in analyzing complex or huge datasets^[310]^ and uncovering the underlying relationships that may not be immediately captured or apparent to human analysts. Recently, researchers have been integrating ML algorithms with optical biosensors to further advance the field. Specifically, ML has been employed for frequency shift analysis of SERS to process large datasets, identify patterns, and recognize subtle spectral changes.^[311]^ For instance, by integrating PCA with a pattern-optimized SERS substrate, Wu et al.^[225]^ achieved breakthroughs in label-free structural analysis, quantification, and species identification of three key AD biomarkers. ML algorithms can also be used for designing highly sensitive SPR biosensors^[312]^ and quantifying the measured biological interactions.^[313]^ Scientists also used ML to extract useful information from experimental data, such as rapid determination of binding affinity,^[314]^ identification of cross-reactive species, and separation of the response signals.^[315]^

## 6. Conclusion and Outlook

In this review, we discussed the emerging role of 2DMs and their mixed-dimensional hybrid nanostructures in advancing label-free optical biosensing. Specifically, we highlighted the unique properties of representative 2DMs, including graphene, TMDCs, MXenes, BP, and hBN, as well as their biosensing applications. We then extended our discussion beyond 2DMs to their mixed-dimensional hybrid nanostructures for label-free optical biosensing, along with the introduction of commonly used synthesis and fabrication strategies. Recent progress in SERS and SPR-based biosensors employing 2DMs and their hybrid architectures was also assessed, with a focus on leveraging synergistic properties for enhanced biosensor performance. Multifunctional platforms based on these hybrid materials were reviewed, including multiplexing, multimodal detection, and the integration of ML for performance optimization, advanced spectral analysis, and data interpretation.

Despite the promising progress, several challenges remain that limit the practical application of 2DMs and their hybrid nanostructures in biosensing. One major issue is the instability and potential toxicity of certain 2DMs. Several strategies can be applied to overcome this limitation, such as surface passivation, encapsulation, or modification of material composition. Through doping, alloying, or developing functional derivatives of the material, improved environmental stability and reduced toxicity can be achieved.

Scalability and reproducibility of hybrid structures also remain challenging. For instance, the performance of SERS substrates with complex integration of mixed-dimensional materials is often difficult to reproduce. In addition, to date, many SPR biosensors with multilayer or complicated structures are still primarily proposed or studied through theoretical modeling or numerical simulations, as these methods are more time-efficient and cost-effective. In practical applications, the majority of commercial SPR biosensors are functionalized relying on Au thin films rather than multilayer structures. Therefore, overcoming the challenges of integration complexity and bridging the gap between theoretical and experimental performance are crucial for the development of biosensors and their translation into real-world applications.

To tackle these issues, researchers are exploring large-area, controllable fabrication techniques that are more robust and capable of producing wafer-scale 2DM heterostructures with high uniformity. Standardized fabrication protocols and reproducible workflows are also essential to ensure consistent quality and performance across biosensors. Additionally, characterization techniques such as Raman mapping, AFM, and XPS should be actively employed to analyze and verify the structural and optical properties of these systems.

Furthermore, a deeper understanding of the underlying physics within multicomponent hybrid nanostructures is essential. Scattering-type scanning near-field optical microscopy (sSNOM), an AFM-based technique, has proven to be highly effective in revealing light–matter interactions and coupling phenomena at the nanoscale. Complementing such advanced techniques with additional characterization methods can provide a more comprehensive understanding of the properties of the studied hybrid nanostructures.

Looking ahead, the future of label-free optical biosensing lies in sensor miniaturization and interdisciplinary integration across physics, materials science, chemistry, optical engineering, and biomedical engineering. The unique properties of 2DMs and their hybrid nanostructures are well-suited to enabling miniaturized and flexible platforms, paving the way toward the “lab-on-a-chip” vision. By leveraging both the flexibility and unique optical properties of 2DMs, wearable label-free optical biosensors could enable non-invasive detection and remote monitoring with high sensitivity and specificity. Integrating ML with biosensing technologies could further enhance system performance, enable rapid and sophisticated analysis of large or multidimensional datasets, and support real-time data interpretation. Interdisciplinary efforts will continue to drive the development of next-generation intelligent biosensors—capable of non-invasive, real-time, remote monitoring for healthcare diagnostics and beyond.

## Figures and Tables

**Table 1.** Overview of different 2DMs and their applications in label-free optical biosensing.

2DMs	Properties/advantages	Synthesis methods	Applications in label-free optical biosensing		Disadvantages	Refs.
Graphene	Zero-bandgap semi-metal, very high electrical conductivity; high tensile strength; excellent thermal conductivity; high optical transparency; excellent biocompatibility; easy surface functionalization	Mechanical exfoliation; CVD; epitaxial growth	SPR	Charge transfer at the graphene-metal thin film interface; modifies the local RI and enhances the electric field intensity	Low intrinsic optical absorption (~2.3% per layer) limits its effectiveness in SPR and SERS; graphene supports plasmons in MIR and terahertz ranges, making it less compatible with biosensing platforms based on visible light	[51, 69, 70, 71]
			SERS/GERS	Charge transfer (strong *π-π* interaction)		
			SEIRA	Enhances IR absorption		
TMDCs	Semiconducting nature; tunable bandgap; layer-dependent PL properties, light absorption in the visible-NIR regime; strong fluorescence quenching capability	Mechanical exfoliation; CVD; MOCVD	SPR	Higher light absorption efficiency (than graphene); strong charge transfer	Degradation and oxidation upon exposure to air; photoinduced oxidation; many TMDCs are hydrophobic, making them less efficient in biomolecule adsorption	[72, 71, 73, 74, 75]
			SERS	Charge transfer; EF increases as the number of layers decreases		
			PL biosensors	PL signals are modulated upon interaction with bound target analytes		
Mxenes	High electrical conductivity; plasmonic behavior in the NIR-MIR regime; surface termination groups can be modified to tune the electrical, optical, and chemical properties; fluorescence quenching capability; hydrophilic	Selective chemical etching of *MAX* phases; liquid-phase exfoliation	SPR	Tunable plasmonic behavior; charge transfer between MXenes and metal thin films; Effective analyte recognition layer	Lack of controllable synthesis methods; challenges in mass production; prone to oxidation and structural degradation, limiting long-term use	[76, 77, 78, 79, 80]
			SERS	Strong affinity capability; strong charge transfer; EF increases as the number of layers increases		
BP	High in-plane anisotropy; high carrier mobility; layer-dependent direct bandgap; broadband optical absorption in the visible-NIR regime; strong fluorescence quenching capability	Exfoliation	SPR	Increases carrier mobility; enhances the electric field intensity at the interface when combined with other 2DMs; charge transfer	Instability in air and water; relatively low EF when used as SERS substrates	[81, 82, 83, 84, 85, 86]
			SERS	Charge transfer; EF increases as the number of layers decreases; polarization-dependent enhancement; minimal spectral interference with NIR excitation, highly desirable for biological samples		
hBN	Large bandgap, electrical insulator; optically transparent (UV-NIR); excellent thermal stability; chemical inertness; smooth surface	Mechanical exfoliation; CVD	SPR	Protective layer or dielectric spacer	Limited sensing capabilities as direct transducer material due to its electrical insulating nature; relatively low EF when used as SERS substrates	[20, 25, 87, 75]
			SERS	Dipole-dipole interactions; no dependence on number of layers; Protective layer		
Antimonene	High carrier mobility; semiconductor; tunable bandgap; larger work function than graphene and other 2DMs; strong spin-orbit coupling; high stability; hydrophilic	Liquid-phase exfoliation; epitaxial growth	SPR	High binding energy enables strong adsorption of analytes	Challenges in scalable synthesis; limited surface functionalization strategies	[88, 71, 89, 90, 91, 92]

**Table 2.** Overview of recently reported 2DMs used as SERS substrates.

Materials	Target analyte		LOD	EF	Other features/functions	Refs.
Metallic 2H-TaS_2_ nanosheets	R6G		3.01 × 10^−18^ m	1.3 × 10^14^	–	[176]
	NBA		4.05 × 10^−21^ m	–		
2D HfTe_2_ nanosheets	R6G		10^−9^ m	2.32 × 10^6^	–	[177]
	Uric acid		0.1 mmol L^−1^	–		
CVD-grown few-layer 1T′-MoTe_2_	*β*-sitosterol		10^−9^ m	–	Reusability	[178]
CVD-grown	2H-MoTe_2_	MB	10^−8^ m	5.4 × 10^8^	–	[179]
	1T’-MoTe_2_			1.8 × 10^7^		
Mono- and bi-layer 1T-MoSSe	R6G		10^−12^ m	6.0 × 10^6^	–	[180]
Ti_3_C_2_ MXene	MB		10^−7^ m	2.9 × 10^6^	–	[163]
	Methyl violet (MV)		10^−6^ m	2.84 × 10^5^		
Highly crystalline monolayer Ti_3_C_2_ nanosheets	Typical environmental pollutants (azo dyes, trichlorophenol, and bisphenol A)		10^−11^ m	3.82 × 10^8^		[194]
Ti_3_C_2_T_x_ nanosheets (with varying thickness)	MB		–	~1.3 × 10^5^ (2 μm-thick)	–	[78]
MXenes (Nb_2_C, Mo_2_C, Ti_2_C, V_2_C, Ti_3_C_2_, Mo_2_TiC_2_, and Ti_3_CN)	R6G		10^−7^ m	–	–	[184]
V_4_C_3_	R6G		10^−7^ m	1.32 × 10^5^	Flexible; rapid molecular enrichment	[185]
V_2_C				0.76 × 10^5^		
Few-layered TiVC nanosheets	R6G		10^−15^ m	3.27 × 10^12^	–	[186]
Ta_4_C_3_	R6G		10^−7^ m	1.51 × 10^5^	–	[188]
	CV		10^−6^ m (0.24 mg kg^−1^)	–		
Nb_4_C_3_	R6G		5 × 10^−7^ m	0.52 × 10^5^		
Nb_2_C nanosheets	MB		10^−8^ m	3 × 10^6^	–	[187]
	MV		10^−6^ m	1.5 × 10^5^		
Ta_2_C nanosheets	MB		10^−6^ m	3.8 × 10^5^		
	MV		10^−7^ m	1.4 × 10^6^		
	SARS-CoV-2 S protein		5 × 10^−9^ m	–		
Few-layer hBN film	MB		–	1.4 × 10^4^	Highly stable hBN (> 7 months)	[192]
	MG			6.4 × 10^3^		
	R6G			1.5 × 10^4^		

**Table 3.** Overview of recently reported metal NPs/2DM hybrids used as SERS substrates.

Materials	Target analyte		LOD	EF	Other features/functions	Refs.
Au NPs/CVD hBN film	MB		–	1.6 × 10^6^	Highly stable hBN (> 7 months)	[192]
Ag NPs/BP-nanosheets	R6G		10^−20^ m	1.01 × 10^11^	Polarization-mapping strategy; combined with ML	[135]
Ag NPs/BP flakes	Sepsis biomarkers: interleukin-3 (IL-3); procalcitonin (PCT)		1000 fm; 100 fm	~1 × 10^14^	–	[197]
Au NPs-Ag NPs/BP nanosheet	4-MBA		4.5 × 10^−10^ m	2.5 × 10^7^	Photocatalytic property (self-cleaning, recyclable); molecular enrichment; flexible	[198]
	Thiram		2.6 × 10^−6^ mg mL^−1^	–		
Ag NPs/Ti_3_C_2_T_x_	R6G		10^−14^ m	3.8 × 10^8^	–	[100]
	Adenosine triphosphate (ATP)		4.27 × 10^−9^ m	–		
	Folate acid (FA)		7.26 × 10^−13^ m	–		
Au NPs/Ti_3_C_2_T_x_	4-MBA		10^−9^ m	–	Antibacterial and photothermal sterilization effects	[153]
	*E. coli*		10^6^ CFU mL^−1^			
	*B. subtilis*		3 × 10^5^ CFU mL^−1^			
Ag NPs/Ti_3_C_2_T_x_	4-MBA		10^−8^ m	3.15 × 10^6^	Long-term stability (1 month)	[162]
	Adenine		10^−8^ m	–		
	Dopamine		5 × 10^−8^ m	–		
Au NPs/Mo_2_C nanosheets	MB		10^−8^ m	2.2 × 10^4^	Long-term stability (~1 month)	[171]
Ag NPs/Ti_3_C_2_T_x_	R6G		10^−9^ m	7.08 × 10^5^	Long-term stability (1 month)	[172]
	Furfural		0.5 mg L^−1^	–		
Ag NPs/Ta_4_C_3_	R6G		10^−8^ m	1.53 × 10^6^	–	[202]
	Pesticide ziram		10^−6^ m	1.35 × 10^4^		
Au NPs/TiVC MXene	R6G		10^−15^ m	8.08 × 10^10^	Flexible; wearable; target enrichment	[201]
	Nicotine		10 nm	–		
	Methotrexate		10^−8^ m			
	Nikethamide					
	6-acetylmorphine					
Ag NPs/Ti_3_C_2_T_x_/PMMA	MB		4.37 × 10^−10^ m	7.14 × 10^6^	Flexible; self-rectification capability	[200]
	SARS-CoV-2 N-protein		3.24 × 10^−9^ mg mL^−1^	–		
Au nanostars/Ta_4_C_3_	PATP		10^−9^ m	–	Flexible; in situ analysis (paper-based SERS)	[204]
	Thiram		10^−7^ m			
Au NPs/TiC	Chlorpromazine (CPZ)		3.92 × 10^−11^ m	10^9^	–	[199]
Au-Ag nanoshuttles/Ti_2_C MXene	Carbendazim (CBZ)		0.01 μM	–	Electrochemical and SERS; combined with ML	[203]
Ag NCs/Ti_3_C_2_T_x_	R6G		6.9 × 10^−11^ m	4.77 × 10^7^	–	[189]
	Ritonavir		5.62 × 10^−7^ mg mL^−1^	–		
	Ibrutinib		1.17 × 10^−6^ mg mL^−1^	–		
Au NPs/MoS_2_ nanosheets	TNT		2 × 10^−7^ m	–	–	[205]
Ag NPs/mesh-like MoS_2_	R6G		10^−8^ m	3.14 × 10^3^	Rapid detection capability in flowing water	[206]
	MG		10^−8^ m	2.77 × 10^2^		
Au NPs/ReSe_2_	R6G		10^−10^ m	3.8 × 10^5^	–	[207]
Au NPs/MoS_2_			10^−10^ m	3.5 × 10^5^		
Au NPs/PdSe_2_			10^−12^ m	4.5 × 10^5^		
Au NPs/CVD graphene/polyester film	R6G		10^−8^ m	10^9^	Flexible; transparent	[208]
	L-tyrosine		10^−8^ m	7.5 × 10^9^		
Ag NPs/graphene	R6G		10^−13^ m	3.03 × 10^11^	–	[210]
Ag NPs/laser-induced graphene	R6G		10^−8^ m	3.19 × 10^5^	–	[211]
	CV		10^−7^ m	–		
	MG		10^−7^ m	–		
Au NPs/GO	RhB		10^−2^ mm	> 7.8 × 10^2^	Combined with ML	[212]
	*Aβ* 1–42		0.0232 ng mL^−1^ (monomer)	–		
	0		.0192 ng mL^−1^ (fibrils)			
Au NPs/GO	R6G		10^−10^ m	3.5 × 10^6^	Flexible	[213]
	Direct Blue 200		10^−10^ m	–		
	Adenine		< 10^−10^ m	–		
Ag NPs/rGO	R6G		10^−7^ m	3.03 × 10^5^	In situ enrichment effect; excellent catalytic property	[214]
Au nanostars/rGO	BaP		0.0028 μg L^−1^	–	–	[215]
Au@Ag NPs-Au nanostars/GO	4-MBA		–	1.2 × 10^8^	Enrichment capability	[221]
	BR		10^−11^ m	–		
Ag NCs/graphene	R6G		10^−12^ m	~1.5 × 10^9^	–	[216]
	POPs	Dichlorodiphenyltrichloroethane (DDT)	~4.13 × 10^−9^ m			
	Fluorene		~4.47 × 10^−9^ m	–		
	Naphthalene		~1.7 × 10^−8^ m	–		
Triangular Ag nanoplates/GO	Adenine		10^−8^ m	1.09 × 10^8^	Flexible	[217]
	*Staphylococcus aureus* (*S. aureus*)		10^2^ CFU mL^−1^	–		
Au NPs/GO	5,5′-dithiobis(2-nitrobenzoic acid) (DTNB)		10^−14^ m	1.01 × 10^5^	Molecular enrichment; multiplex capability	[218]
	Pesticides	Fenthion	38.01 ng mL^−1^	–		
	Phoxim		8.13 ng mL^−1^	–		
	Isocarbophos		48.97 ng mL^−1^	–		
	Thiram		8.74 ng mL^−1^	–		
Au@Ag nanostars/GO	Thiophenol (TP)		0.05 nm	3.2 × 10^8^	Multiplex capability	[219]
	Pesticides	Ziram	10 pm	–		
	Phorate		50 pm	–		
	Triazophos		100 pm	–		
	Azinphos-methyl		100 pm	–		

**Table 4.** Overview of 0D-2D hybrids featuring complex or specially engineered architectures for SERS sensing.

Materials	Target analyte		LOD	EF	Other features/functions	Refs.
Gr MFs/wrinkled MoS_2_	RhB		5 × 10^−11^ m	2.96 × 10^7^	Molecular enrichment; long-term stability (>50 days)	[190]
MoS_2_@Ag NFs/rGO	MB		5.2 × 10^−11^ m	8.6 × 10^6^	Photocatalytic capability (in situ degradation; recyclable); molecular enrichment; multiplex capability	[229]
	Melamine		4.72 × 10^−9^ M	–		
	Vanillin (VA)		5.58 × 10^−9^ M			
	BPA		7.71 × 10^−8^ M			
TMD (MoS_2_ and WS_2_) NDs/graphene vdW	R6G		5 × 10^−11^−5 × 10^−12^ m	–	–	[222]
			
Au NPs/WS_2_ NDs/graphene	R6G		10^−12^ m	–	–	[191]
WS_2_ nanodisks-MoS_2_ nanodisks/graphene	R6G		~5 × 10^−13^ m	–	–	[230]
Au@Cu_2_O nanotriangles/Ti_3_C_2_T_x_ ultrathin nanosheets	MB		10^−12^ m	2.4 × 10^9^	Structure-adjustable; photocatalytic potential	[164]
MOF@Ag NFs/Ti_3_C_2_T_x_ ultrathin nanosheets	MB		6.99 × 10^−8^ m	–	Flexible; molecular enrichment	[231]
	*E. coli*		< 9 × 10^2^ CFU mL^−1^	–		
ZnO QDs/Ti_3_C_2_ MXene	4-MPY		10^−7^ m	4.6 × 10^4^	Flexible; photocatalytic capability (self-cleaning, recyclable); in situ detection; long-term stability (60 days)	[232]
Fe_3_O_4_@Au NPs/GO	PAHs	BaP	3.8 μg L^−1^	–	Photocatalytic capability (in situ degradation); long-term stability (> 130 days)	[223]
		Phenanthrene	10 μg L^−1^	–		
		Anthracene	10 μg L^−1^	–		
		Pyrene	1 μg L^−1^	–		
		Fluoranthene	1 μg L^−1^	–		
Graphene/Au nanopyramids	R6G		10^−13^ m	1.5 × 10^10^	Wafer-scale; multiplex capability; PCA	[225]
	Tau & phospho-Tau (P-Tau) proteins		10^−15^ m	–		
	A*β*42 polypeptide		10^−14^ m	2 × 10^8^		
Au NPs/graphene/Cu cone cavities	MG		10^−9^ mol L^−1^	–	Long-term stability	[226]
	Paraquat		10^−7^ mol L^−1^	–		
Graphene/Au NPs/rectangular pyramid PMMA hybrids	Paraquat		10^−8^ m	–	Flexible; in situ detection; long-term stability (30 days)	[227]
	2,4-d		10^−6^ m	–		
Ti_3_C_2_ nanosheets/Au-Ag bimetallic nanocuboids	CV; MG; MB		10^−12^ m	–	Multiplex capability	[228]

**Table 5.** Overview of recently reported 1D–2D hybrid nanostructures used as SERS substrates.

Materials	Target analyte	LOD	EF	Other features/functions	Refs.
Monolayer hBN/Ag NRs	R6G	7.9 × 10^−10^ m	1.26 × 10^8^	Recyclable; long-term stability	[234]
	BR	2.5 × 10^−8^ m	–		
Ti_3_C_2_ MXene/Ag NRs	CV	2.48 × 10^−11^ m	–	Multiplex capability; long-term stability	[235]
	PCB-77	2.43 × 10^−10^ m	–		
	PCB-3	2.14 × 10^−9^ m	–		
Ti_3_C_2_T_x_/ZnO NRs	R6G	10^−11^ m	1.49 × 10^7^	–	[236]
	miRNA	10^−6^ m	–		
WO_3-x_ NWs/WSe_2_	MB	5 × 10^−18^ m	5.0 × 10^11^	–	[237]

**Table 6.** Overview of recently reported multicomponent nanohybrids with mixed dimensions for SERS sensing.

Materials	Target analyte		LOD	EF	Other features/functions	Refs.
hBN/graphene/Ag NPs	R6G		10^−6^ m	3.65 × 10^5^	Reusable	[248]
GO/Ag/PS nanospheres	2-Naphthalenethiol (2-NAT)		2.8 × 10^−8^ m	2.27 × 10^6^	Multiplex capability; molecular enrichment	[174]
	Melamine		2.81 × 10^−10^ m	–		
	Dicyandiamide		6.03 × 10^−9^ m	–		
Ag nanostars-MWCNT/GO	4-MPY		10^−8^ m	–	Analyte enrichment	[249]
	PS (50–500 nm)		5 × 10^−5^ mg mL^−1^			
	PMMA nanoplastics (50 nm)		5 × 10^−4^ mg mL^−1^			
Au NPs/GO/Ag NWs	PAHs	Pyrene	10^−8^ m	1.48 × 10^5^	Multiplex capability	[238]
		Anthracene	10^−8^ m	–		
		Phenanthrene	10^−8^ m	–		
		BaP	10^−7^ m	–		
Au/graphene/Ag/ZnO	R6G		10^−13^ m	5.68 × 10^7^	Molecular enrichment; photocatalytic degradation capability (reusable)	[173]
			
Ag NPs/graphene/Cu	R6G		10^−14^ m	8.86 × 10^8^	Flexible	[250]
Ag NPs/graphene/WTe_2_	R6G		10^−13^ m	1.34 × 10^12^	–	[239]
	CV		10^−10^ m	4.76 × 10^10^		
	MB		10^−8^ m	4.53 × 10^8^		
	MG		10^−7^ m	5.19 × 10^7^		
Au NPs/MoS_2_/ Ti_3_C_2_ MXene	miRNA-182		6.61 am	4.8 × 10^8^	Self-internal standards (synergistic calibrated strategy)	[251]

**Table 7.** Summary of recent Kretschmann SPR biosensors with 2DMs and their hybrid nanostructures.

Materials	Analyte		Sensitivity [deg RIU^−1^]	Quality factor [RIU^−1^]	Refs.
Graphene/Au/N-FK51A prism	Glucose (25–175 mgdL^−1^)		275.15	76.2	[264]
	Gas (RI: 1.0000–1.0007)		92.1	230.2	
Graphene/AlN/Ag/TiO_2_/BK7 prism	Infected plasma		138.46	–	[266]
	Infected platelet		163.63		
	Infected hemoglobin		182.85		
Thiol-tethered ssDNA/graphene/BaTiO_3_/Ag/TiO_2_/CaF_2_ prism	SARS-CoV-2 virus		433.63	136.79	[267]
			
Graphene nanoribbons/SiO_2_/graphene/Si	RI	1.33	36 401.1 mV RIU^−1^	21.84	[256]
		1.34	40 676.5 mV RIU^−1^	24	
		1.35	40 918.2 mV RIU^−1^	23.74	
		1.36	41 160 mV RIU^−1^	23.69	
Au NRs/Antimonene/Au/prism	RI		171	–	[88]
	miRNA-21 and miRNA-155		10 am		
Antimonene/Au/BK7 prism	RI		181.9	26.400	[142]
Graphene/Au/BK7 prism			147.95	30.370	
Au/BK7 prism			144.45	33.671	
Antimonene/BaTiO_3_/Ag/BaF_2_ prism	RI		303.83	50.39	[147]
	Hemoglobin concentration		0.045 deg/gL^−1^ (concentration sensitivity)	–	
Antimonene/ZnO/Ag/SF11 prism	RI		105.72	14.89	[146]
WS_2_/BaTiO_3_/Ag/ZnO/BK7 prism	RI		180 (1L); 235.00 (2L)	63.51	[257]
MoS_2_/BaTiO_3_/Ag/ZnO/BK7 prism			174 (1L); 202.00 (2L)	34.17	
Graphene/BaTiO_3_/Ag/ZnO/BK7 prism			157 (1L); 162.50 (2L)	70.96	
WSe_2_/Si/Ag/prism	Urea in blood (1 g dL^−1^)		373.49	–	[270]
WS_2_/GaSe/Cu/BK7 prism	RI (dengue virus)		303.28	97.51	[271]
WSe_2_/Al/Ag/CaF_2_ prism	RI		210	225.80	[273]
Ti_3_C_2_T_2_ (T=O, OH, F) MXene/Au/Cr/BK7 prism	RI		150.131 (Ti_3_C_2_F_2_)	–	[80]
Ti_3_C_2_T_x_/Ag/CaF_2_ prism	RI		350.87	42.10	[276]
Ti_3_C_2_T_x_/Au/Ag/MgO/FK51A prism	RI		236	~63	[79]
Ti_2_C MXene/Au/prism	RI		3579.6 nm RIU^−1^	26.168	[128]
	Pb^2+^		79.2 ng L^−1^ (LOD)	–	
	Cr^2+^		56.5 ng L^−1^ (LOD)		
	Hg^2+^		92.8 ng L^−1^ (LOD)		
BP/Ni/SiO_2_/Cu/BK7 prism	RI		479.10	102.19	[279]
BP/Ag/BaTiO_3_/Ag/BK7 prism	RI		360	72.6	[281]
BP/Au/BaTiO_3_/Au/BK7 prism			299	39.6	
BP/Cu/BaTiO_3_/Cu/BK7 prism			378	145.1	
Graphene/BP/Ag/TiO_2_/CaF_2_ prism	RI (SARS-CoV-2)		390	92.86	[283]
Graphene/BP/Cu/CaF_2_ prism	RI (SARS-CoV-2 Omicron)		410	94.25	[258]
Antimonene/borophene/Au/BK7 prism	RI; biomolecules (DNA/RNA)		206.26	–	[144]
Antimonene/BP/Si/Al/SF10 prism	RI		202.37	28.86	[143]
	DNA hybridization		2.8 pm	0.037 nm^−1^	
Antimonene/BP/Ni/Cu/BK7 prism	RI		446.90	93.10	[259]
Graphene/MoSe_2_/Ag/Ti/BK7 prism	RI		215.5	–	[286]
Graphene/Ag/Ti/BK7 prism	CEA in PBS		133.33	107.52	[288]
Graphene/MXene/Ag/Ti/BK7 prism			140.27	62.62	
Graphene/MoS_2_/Ag/Ti/BK7 prism			144.72	62.38	
Ti_3_C_2_T_x_/graphene/Ag/glass prism	RI (1.33–1.36)		214.20	–	[289]
Antimonene/Ti_3_C_2_T_x_/Au/TiO2/BK7 prism	RI		224.26	19.06	[290]
Ti_3_C_2_T_x_/BP/Ni/Cu/BK7 prism	RI		304.47	57.85	[291]
Ti_3_C_2_T_x_/BP/Si/Ag/BK7 prism	RI		264	41.25	[260]
hBN/LiF/Ag/SF11 prism	RI		1000	225	[261]
Ti_3_C_2_T_x_/MoS_2_/optical waveguide layer/coupling dielectric layer/S-BSL7M prism	RI		86.13	281.74	[292]
Blue phosphorus-MoS_2_/Au/PtSe_2_/CaF_2_ prism	RI (1.33–1.36)		240.54	29.23	[293]
WS_2_/HfSe_2_/BP/Ag/BAK1 prism	RI: 1.33–1.39 (volatile organic compounds (VOCs))		227.14	–	[294]
Ti_3_C_2_T_x_/MoO_3_/MoS_2_/Ag/BK7 prism	RI (glucose solution)		227.08	35.09	[295]

**Table 8.** Recent fiber-optic SPR biosensors with 2DM hybrid nanostructures.

2DM hybrid nanostructures	Analyte	Sensitivity		LOD	Quality factor [RIU^−1^]	Refs.
Graphene/BP/Ag	RI	4050 nm RIU^−1^		–	39.70	[297]
MoS_2_/BP/Ag		3950 nm RIU^−1^			40.25	
WS_2_/BP/Ag		3975 nm RIU^−1^			47.89	
MoSe_2_/BP/Ag		3975 nm RIU^−1^			41.40	
WSe_2_/BP/Ag		4000 nm RIU^−1^			47.62	
Antimonene/graphene/Au RI (cancerous cells)	Skin	7.3465 μm RIU^−1^		7.2 × 10^−5^ RIU	131.1525	[145]
	Cervical	10.9250 μm RIU^−1^				
	Blood	11.8914 μm RIU^−1^				
	Adrenal	15.2414 μm RIU^−1^				
Graphene/MoS_2_/Au/Cr	RI	8631.54 nm RIU^−1^		–	–	[298]
	Glucose (0–500 mg dL^−1^)	12 593.06 nm RIU^−1^				
Nb_2_CT_x_/Au NPs (S-tapered)	Tyramine	34 pm μm^−1^		0.35 μm	–	[300]
Nb_2_CT_x_/Au NPs (W-shaped)	Tyramine	0.0385 nm μm^−1^		6.96 μm	–	[301]
Ti_3_C_2_ Mxene/Au NRs	Carbonic anhydrase IX (CAIX) protein	Pure buffer	1.46 nm/log_10_ m	13.8 zm	–	[299]
		30% serum solution	0.986 nm/log_10_ m	0.19 am		
Ti_3_C_2_ Mxene/Au NRs	Acetylcholine	0.04521 nm μm^−1^		4.42 μm	–	[302]
